# Cancer research in Saudi Arabia: A cross-sectional mapping study of historical growth, thematic analysis, collaboration patterns, and future directions

**DOI:** 10.1097/MD.0000000000044757

**Published:** 2025-09-26

**Authors:** Jobran M. Moshi, Siddig Ibrahim Abdelwahab, Manal Mohamed Elhassan Taha, Osama Albasheer, Saleh M. Abdullah, Abdullah Farasani, Mohammed A. Jeraiby, Humaid Obaid Al-Shamsi, Ahlam Mohammed S. Hakami, Nizar A. Khamjan, Saeed Alshahrani, Ahmad Assiri, Hussam M. Shubaily, Khaled A. Sahli

**Affiliations:** a Department of Applied Medical Sciences, College of Nursing and Health Sciences, Jazan University, Jazan, Saudi Arabia; b Health Research Centre, Jazan University, Jazan, Saudi Arabia; c Department of Family and Community Medicine, Faculty of Medicine, Jazan University, Jazan, Saudi Arabia; d Department of Basic Medical Science, Faculty of Medicine, Jazan University, Jazan, Saudi Arabia; e Burjeel Cancer Institute, Burjeel Medical City, Abu Dhabi, United Arab Emirates; f Department of Medical Oncology, Dana-Farber Cancer Institute, Harvard Medical School, Boston, MA; g College of Medicine, Ras Al Khaimah Medical and Health Sciences University, Ras Al-Khaimah, United Arab Emirates; h Gulf Medical University, Ajman, United Arab Emirates; i Emirates Oncology Society, Dubai, United Arab Emirates; j College of Medicine, University of Sharjah, Sharjah, United Arab Emirates; k Department of Obstetrics and Gynecology, Faculty of Medicine, Jazan University, Jazan, Saudi Arabia; l Department of Pharmacology and Toxicology, College of Pharmacy, Jazan, Saudi Arabia; m Department of Dermatology, Faculty of Medicine, Jazan University, Jazan, Saudi Arabia; n Department of Basic Medical Sciences (Pathology), Faculty of Medicine, Jazan University, Jazan, Saudi Arabia; o Security Forces Hospitals Program, General Directorate of Medical Services, Ministry of Interior, Riyadh, Saudi Arabia.

**Keywords:** bibliometric analysis, cancer research, research growth, Saudi Arabia, thematic trends

## Abstract

Cancer is a growing health burden in Saudi Arabia, necessitating targeted research to improve its prevention, diagnosis, and treatment. This study aims to map Saudi Cancer Research (SCR) trends, productivity, thematic evolution, collaboration patterns, and emerging research areas. A cross-sectional bibliometric analysis based on Strengthening the Reporting of Observational Studies in Epidemiology guidelines was conducted using the Scopus database. A multistep Medical Subject Headings-based keyword strategy identified cancer-related publications affiliated with Saudi institutions. The study comprised 4 parts: (1) global cancer research involving Saudi affiliations (N = 40,180; 1961–2024), (2) collaborative Saudi-international articles (N = 18,145; 2020–2024), (3) Saudi-only research outputs (N = 9319; 1961–2024), and (4) original articles produced independently by Saudi institutions (N = 4240; 2020–2024). Data were analyzed using Bibliometrix, VOSviewer, and SPSS for productivity, keyword co-occurrence, author impact (*h*-index, *m*-index, and *g*-index), and thematic clustering. Between 1961 and 2024, SCR exhibited exponential growth, increasing from single-digit annual publications to 5707 in 2024. Original research dominated (80.2%), and collaboration spanned 124 countries, with Egypt, India, and the United States as top partners. King Saud University led the output and funding. Thematic mapping has revealed a shift from foundational topics (e.g., chemotherapy and inflammation) to advanced themes such as molecular docking, deep learning, and drug discovery. From 2020 to 2024, over 58% of SCR was produced, reflecting strong national prioritization and increasing engagement in cutting-edge, multidisciplinary, and collaborative cancer research. Cancer research in Saudi Arabia has grown substantially over the decades, both in terms of volume and thematic diversity. This multilayered bibliometric assessment highlights key contributors, evolving research directions, and gaps to inform future national cancer research priorities and innovation strategies.

## 1. Introduction

Cancer is a disease caused by cancer cell proliferation, spread, resource utilization, metabolite synthesis, tissue disruption, and co-option of normal noncancerous cells that interfere with normal biological functioning, resulting in pain, organ failure, and cancer-related disorders, such as cachexia.^[1]^ Cancer not only affects an individual’s physical health but also has a profound impact on the mental well-being and emotional stability of their family and friends.^[2]^ Cancer kills 10 million people worldwide each year, demonstrating its prevalence and mortality. Cancer has a massive economic impact, including direct medical costs, lost productivity from illness, and early mortality, as well as emotional and psychological repercussions on patients and their families.^[3]^

Cancer is the second leading cause of mortality worldwide, accounting for over 9.6 million deaths in 185 countries.^[4]^ Risk factors include tobacco and alcohol consumption, unhealthy diet, physical inactivity, viral infections, bacterial infections, urban air pollution, ionizing radiation, and indoor smoke. Cancer is expected to continue rising to 21.4 million deaths by 2030 due to demographic changes in the population. Preventive and early detection strategies can help increase survival rates.^[5]^ In 2018, Saudi Arabia had 10,518 cancer deaths and 24,485 new cases, with common cancers including breast cancer,^[6]^ colorectal cancer (CRC),^[7]^ and prostate.^[8]^ The risk factors for CRC include genetic, environmental, age, sex, and inflammatory conditions.^[7]^ The prevalence of brain cancer in Saudi Arabia is low, accounting for 2.0% to 3.2% in females and males.^[9]^ Kidney cancer is more common, with an increasing trend owing to various risk factors.^[10]^ Thyroid carcinoma is more common among females, with a prevalence of 10.1%.^[11]^

Bibliometric analysis is a vital tool for understanding research trends, collaboration networks, and thematic evolution, offering valuable insights to guide future research directions and policy-making.^[12,13]^ While existing studies in Saudi Arabia, such as those by Al-Kahtani et al (2023) on breast cancer, Elwali et al (2024) on pancreatic cancer, Haq et al (2017) on oncology research output from a single institution, and Patil et al (2020) on oral cancer, have provided important findings, they are limited by their narrow scope.^[6,8,14,15]^ These studies are restricted to certain types of cancer or even certain institutions, are outdated, and do not adequately address the overall theme of cancer research development in Saudi Arabia, networking, and international relevance regarding cancer research. The limitations identified above were compensated for in the present work by conducting a synergistic bibliometric analysis of cancer research in Saudi Arabia (SCR), its derivatives from 1961 to the present, key authors, gaps, and even collaborative networks within the cancer research field. The first part of the study examines the history of SCR growth, while the second part analyzes SCR growth from 2020 to 2024 with a special focus on the conceptual structure and emerging trends, with the aim of enhancing national research policies while also making global contributions toward cancer prevention, diagnosis, and treatment. The third section assessed Saudi Arabia’s independent contributions to cancer research, focusing on publications solely from Saudi institutions. It identifies key contributors, dominant areas, emerging trends, and gaps; offers insights to strengthen national research output; and addresses regional cancer challenges.

## 2. Methods

### 2.1. Database selection

The Scopus database was used to collect bibliographic data pertinent to the SCR. Scopus was established by Elsevier in 2004. As stated by Elsevier, Scopus is managed by a group of experts and offers the best comprehensive view of the global research output. The primary purpose of Scopus is to create a comprehensive database of high-quality research publications.^[16]^ In contrast, Scopus’s goal of being situated midway between Elsevier and Clarivate Analytics emphasizes quality instead of quantity, which was the focus of WoS in the first place.^[16,17]^ Bibliometric evaluations situate these publications inside the database, which relies on coverage and represents the extent of their underlying databases, as coverage fundamentally determines the inclusion in the research.^[12,13]^

### 2.2. Search strategy

**Figure 1.**
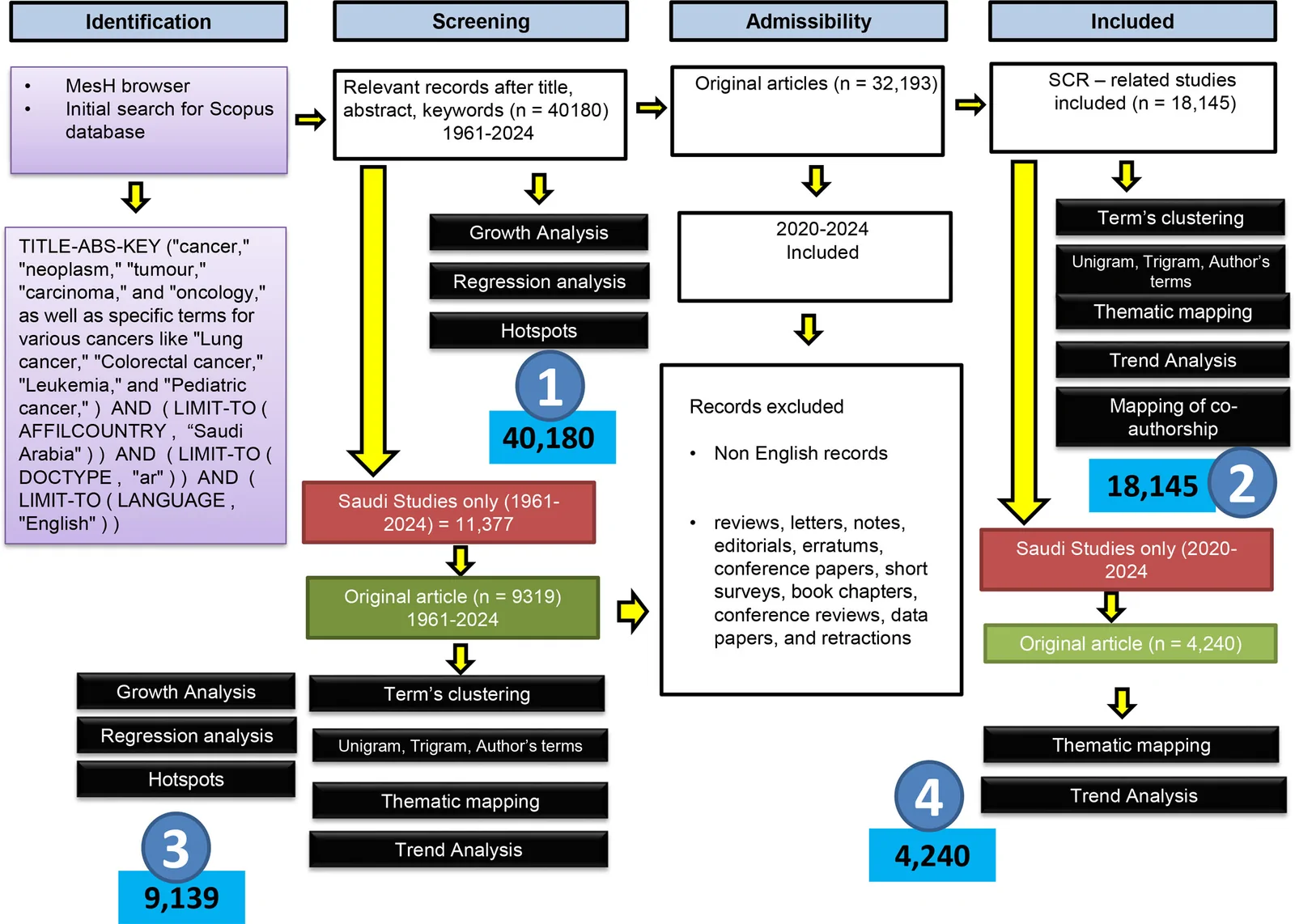
Search strategy from the Scopus database. The data extraction process was designed to meet the requirements of the 4 parts of the study. The strategy involved a systematic approach using Scopus to identify and refine relevant documents, ensuring alignment with the study’s objectives. MeSH = Medical Subject Headings, SCR = Saudi Cancer Research.

A multistage search approach employing the Scopus database^[16]^ was used to find pertinent papers for a bibliometric study on SCR.^[16]^ First, a thorough collection of cancer and oncology-related keywords, including general terms like “cancer,” “neoplasm,” “tumor,” “carcinoma,” and “oncology,” as well as specific terms for various cancers like “Lung cancer,” “Colorectal cancer,” “Leukemia,” and “Pediatric cancer,” among others (Data S1, Supplemental Digital Content, https://links.lww.com/MD/Q99). The Medical Subject Headings database^[18]^ helped to produce keywords. The first search yielded 7,162,715 studies worldwide.^[18]^ Subsequently, the findings were filtered by affiliation nation to concentrate on Saudi Arabia, reducing the dataset to 40,180 papers (first study). Limiting the document type to articles and language to English helped further improve the results (32,193 documents). Written English language material was also included. Precise interpretation, improved data availability, consistency, comparability, relevance to the research inquiry, and encouragement of reproducibility depend on excluding non-English publications and retaining only original papers in bibliometric analysis. By focusing on English articles, researchers can overcome language barriers, access a large body of literature, maintain analytical consistency, match study parameters, and allow validation by the scientific community. A publication year filter was used to concentrate on current research trends, thereby limiting the dataset to papers released between 2020 and 2024 and producing 18,424 findings (second study). Data cleaning was performed in the second section to eliminate duplicates and pointless items, thereby producing a final dataset of 18,145 documents for the study. All articles including foreign affiliations were eliminated to ensure that SCR data (third study) entirely focused on Saudi Arabia, thereby keeping only those whose authors were linked only to Saudi institutions. With no temporal constraints, this dataset reflects a complete collection of Saudi-specific contributions to cancer research, thereby guaranteeing a targeted study of the nation’s autonomous production. This method enables thorough awareness of the local research scene, thereby stressing the successes and contributions motivated by Saudi-based researchers and institutions. After data extraction in October 2024, the dataset (Data S2, Supplemental Digital Content, https://links.lww.com/MD/Q100) was exported in 2 formats, CSV and BibTeX, for an additional study. This method guaranteed a thorough yet concentrated dataset fit for a thorough bibliometric examination. Figure 1 shows the comprehensive procedure for the data selection, search, and analysis.

### 2.3. Document type classification and inclusion criteria

To ensure clarity regarding the types of publications analyzed, we classified all retrieved records from the Scopus database according to their document type. The current study was conducted in 4 parts (Fig. 1), each using refined inclusion criteria to ensure the consistency and quality of the analyzed data. In Study 1, an initial search retrieved 40,180 cancer-related documents (1961–2024) from at least one Saudi affiliation. These include various document types. For Study 2, the dataset was narrowed down to 18,145 original articles published between 2020 and 2024 with international collaboration, excluding reviews, letters, notes, editorials, conference papers, and non-English records. In Study 3, the focus was on Saudi-only contributions, yielding 9319 original research articles from 1961 to 2024 without international collaboration. Study 4 further refined this to 4240 original articles from Saudi institutions, published between 2020 and 2024. This stepwise approach ensured a rigorous and high-quality dataset suitable for bibliometric analysis. This classification allowed for a nuanced understanding of the research contributions by type and ensured methodological transparency.

### 2.4. Data analysis and visualization

Research performance was assessed using the integrated analyzer of the Scopus platform, which can quantify the yearly research output and citations. Bibliographic mapping was performed using Bibliometrix and VOSviewer tools (Leiden, The Netherlands).^[19,20]^ The software program was used for the design and graphical representation of the bibliometric networks. These networks may be created through coauthorship links, and may include journal articles, authors, and even particular articles. The program uses text-mining techniques for the design and graphical representation of co-occurrence networks built from significant terms collected from scientific articles. The Bibliometrix interface in the R package (Biblioshiny) was used to generate the results of this study. VOSviewer is a software application that is used for the construction and graphical representation of bibliometric networks. Such networks can in turn be formed from cited references, bibliographic coupling, co-citation, and coauthorship links among journals, authors, or single titles. VOSviewer employs text mining techniques to build and visualize co-occurrence graphs of important terms extracted from scientific papers.

### 2.5. Statistical analysis

**Figure 2.**
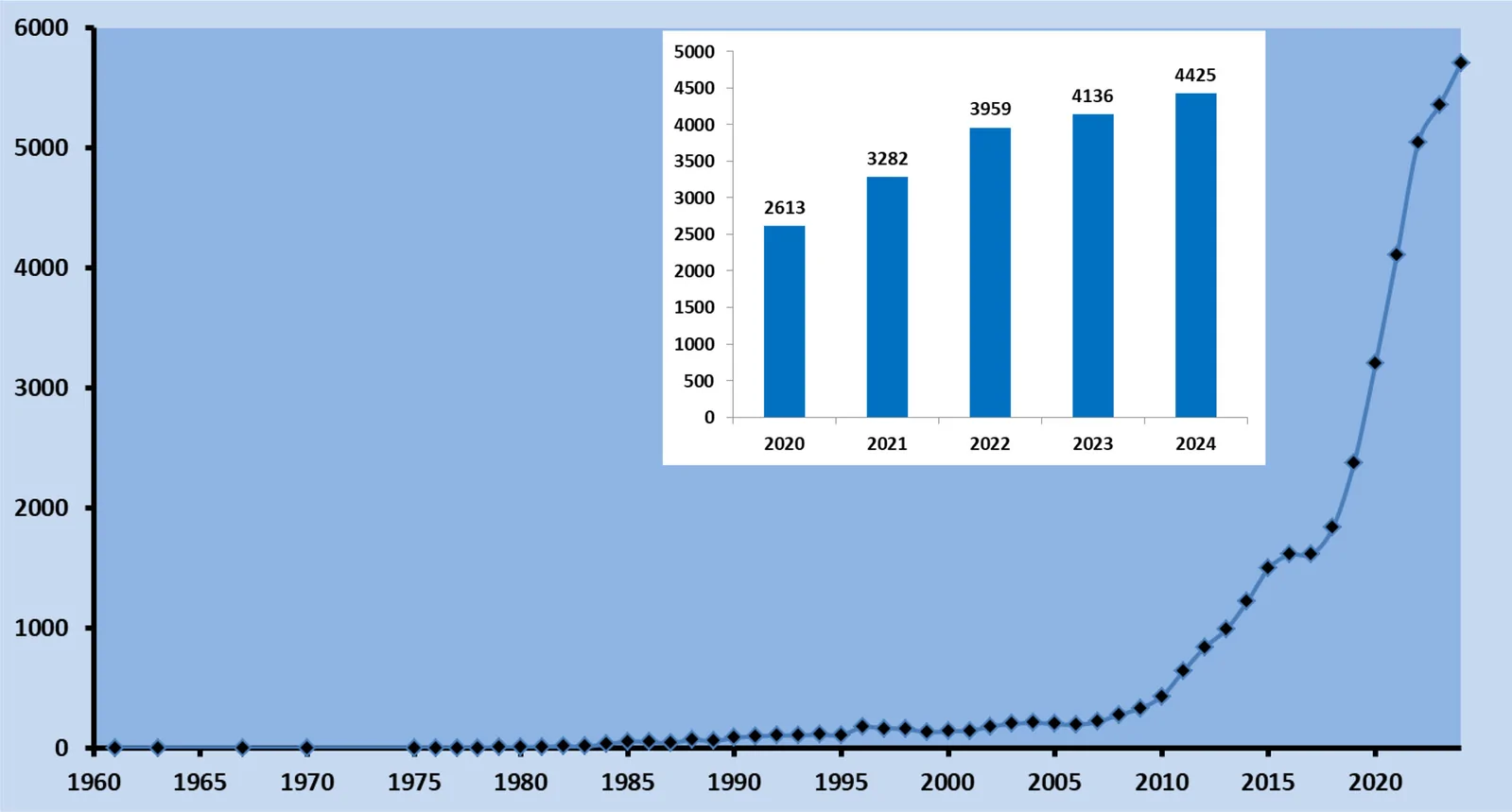
Annual growth of cancer research publications in Saudi Arabia (1961–2024), showing minimal activity (1961–1999), steady growth (2000–2014), accelerated output (2015–2019), and exponential surge (2020–2024), with 58% of total publications produced in recent years. Embedded figure is for the last 5 years (2020-2024).

Statistical analyses were performed using the IBM SPSS version 23 (IBM Corporation, Armonk). Annual production trends in cancer research were assessed using both exponential and polynomial regression models depending on the nature of the dataset in each part of the study. Regression coefficients (*R*^2^) and *P*-values were calculated to evaluate goodness of fit and statistical significance. Statistical significance was set at *P* < .05. The regression results were primarily applied to assess the temporal evolution of research output in Section 3.1 and Section 3.3.2 (Saudi-only research growth) and their corresponding figures (Figs. 2 and 8). These models provide quantitative insights into the strengths and trajectories of publication trends over time.

**Figure 3.**
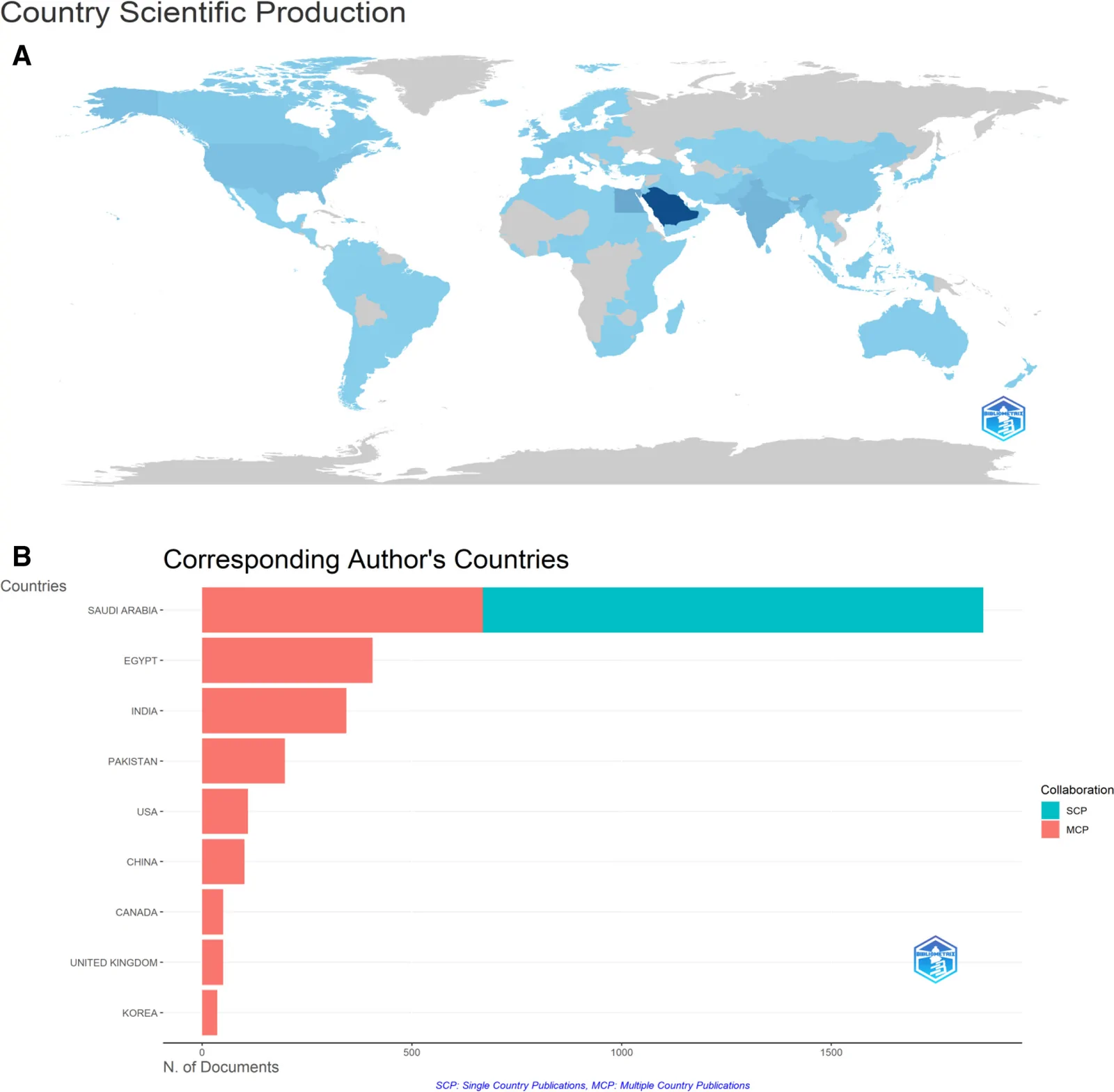
Global landscape and collaboration patterns in KSA cancer research. (A) Top collaborating countries with KSA, where the density of blue color represents the number of articles, led by Egypt (2433), India (1627), and the United States (942). (B) Collaboration ratios: SCP (64%, green) indicates single-country publications, while MCP (36%, orange) represents multi-country collaborations, highlighting KSA’s balanced research efforts. KSA = Kingdom of Saudi Arabia.

**Figure 4.**
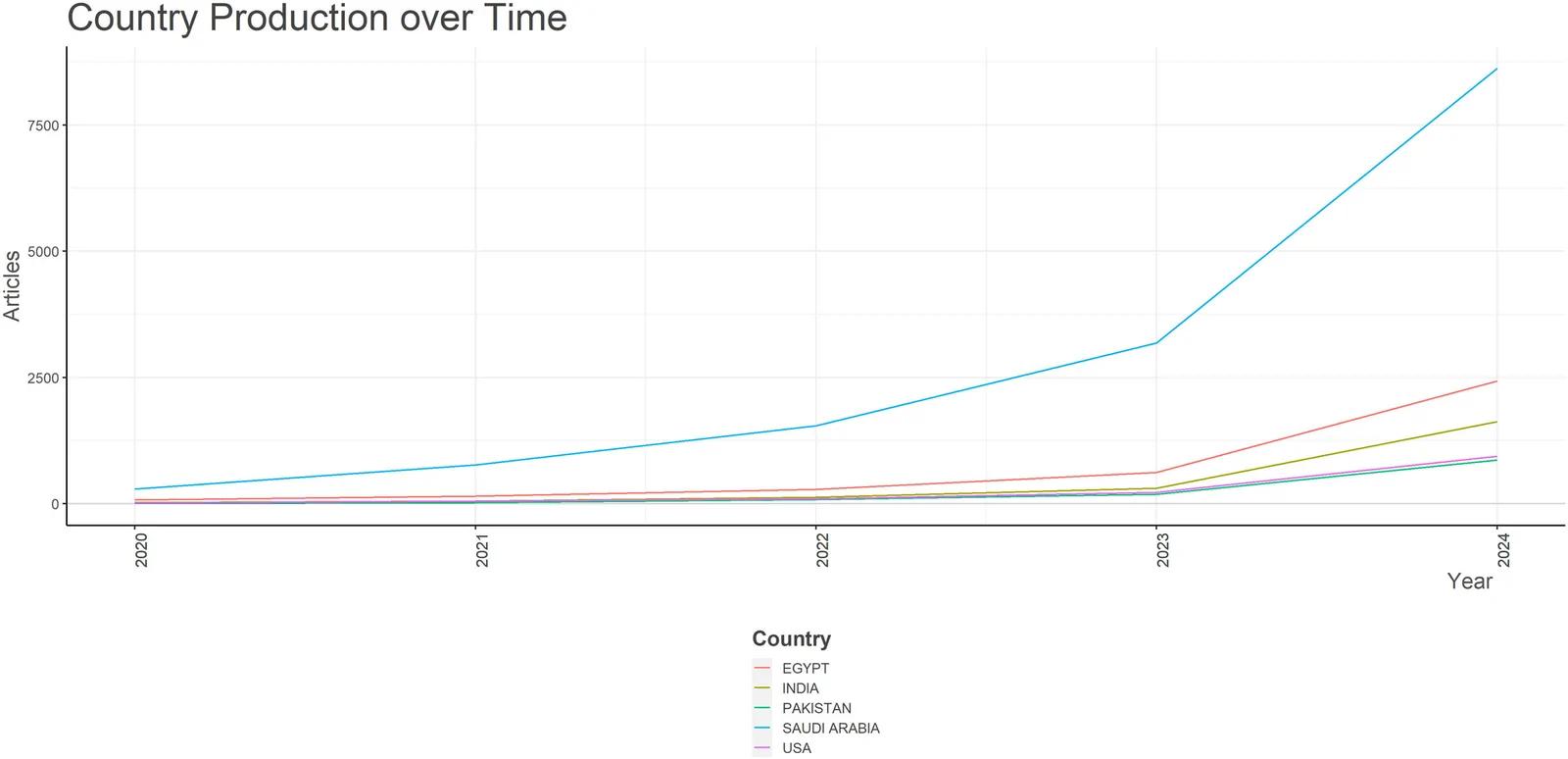
Country production over time.

**Figure 5.**
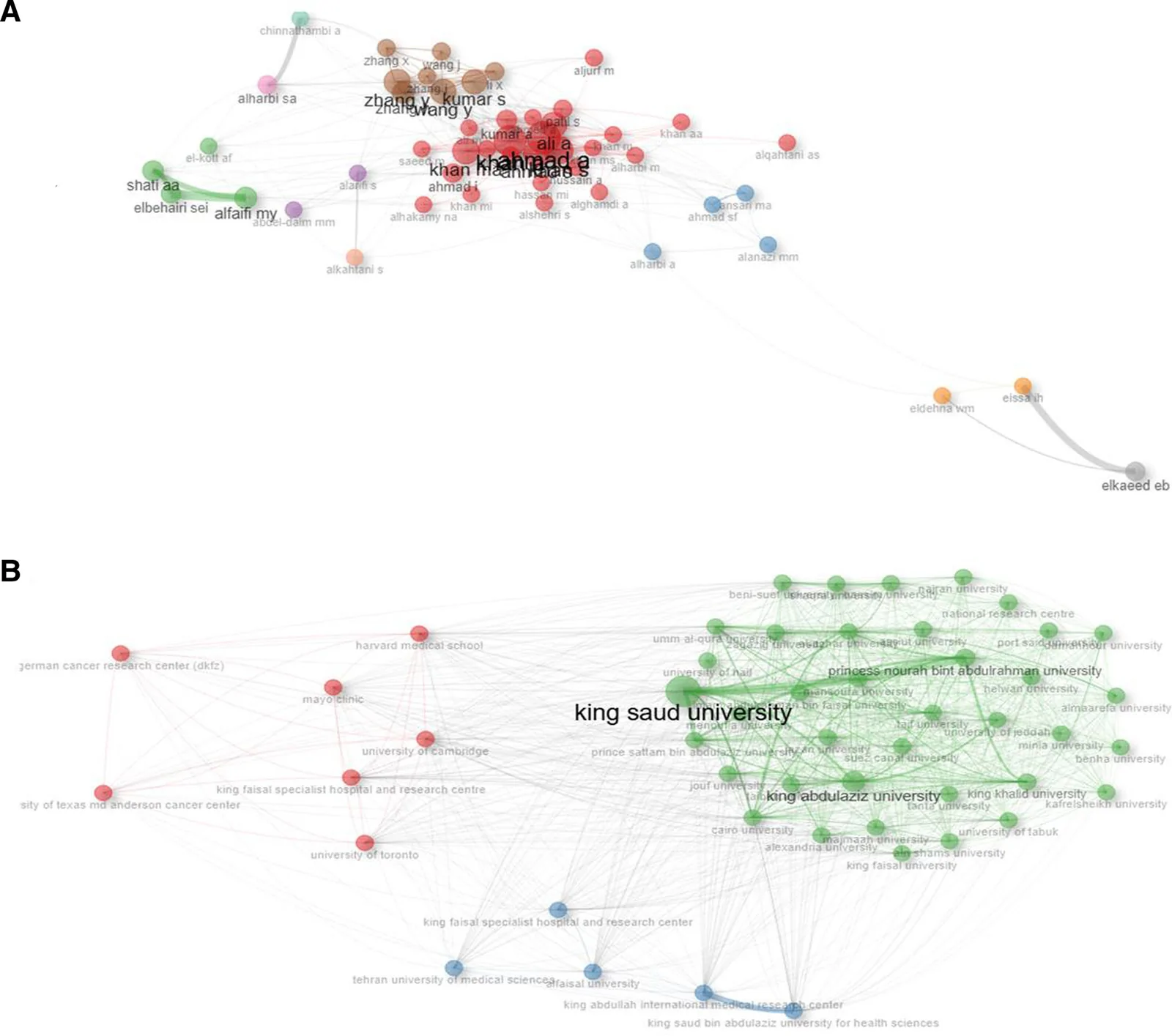
Collaboration networks in KSA cancer research. (A) Author collaboration network: nodes represent authors, with node size indicating collaboration strength, and colors distinguishing clusters. Betweenness centrality highlights key authors, such as Ahmad A and Khan M, driving collaborations. (B) Institutional collaboration network: nodes represent institutions, with node size reflecting collaborative strength. Clusters include global institutions (e.g., University of Toronto) and key local contributors (e.g., King Saud University, King Faisal Specialist Hospital and Research Centre). The analysis reveals strong national collaborations alongside significant international partnerships, emphasizing the interconnected research landscape in KSA cancer research. KSA = Kingdom of Saudi Arabia.

**Figure 6.**
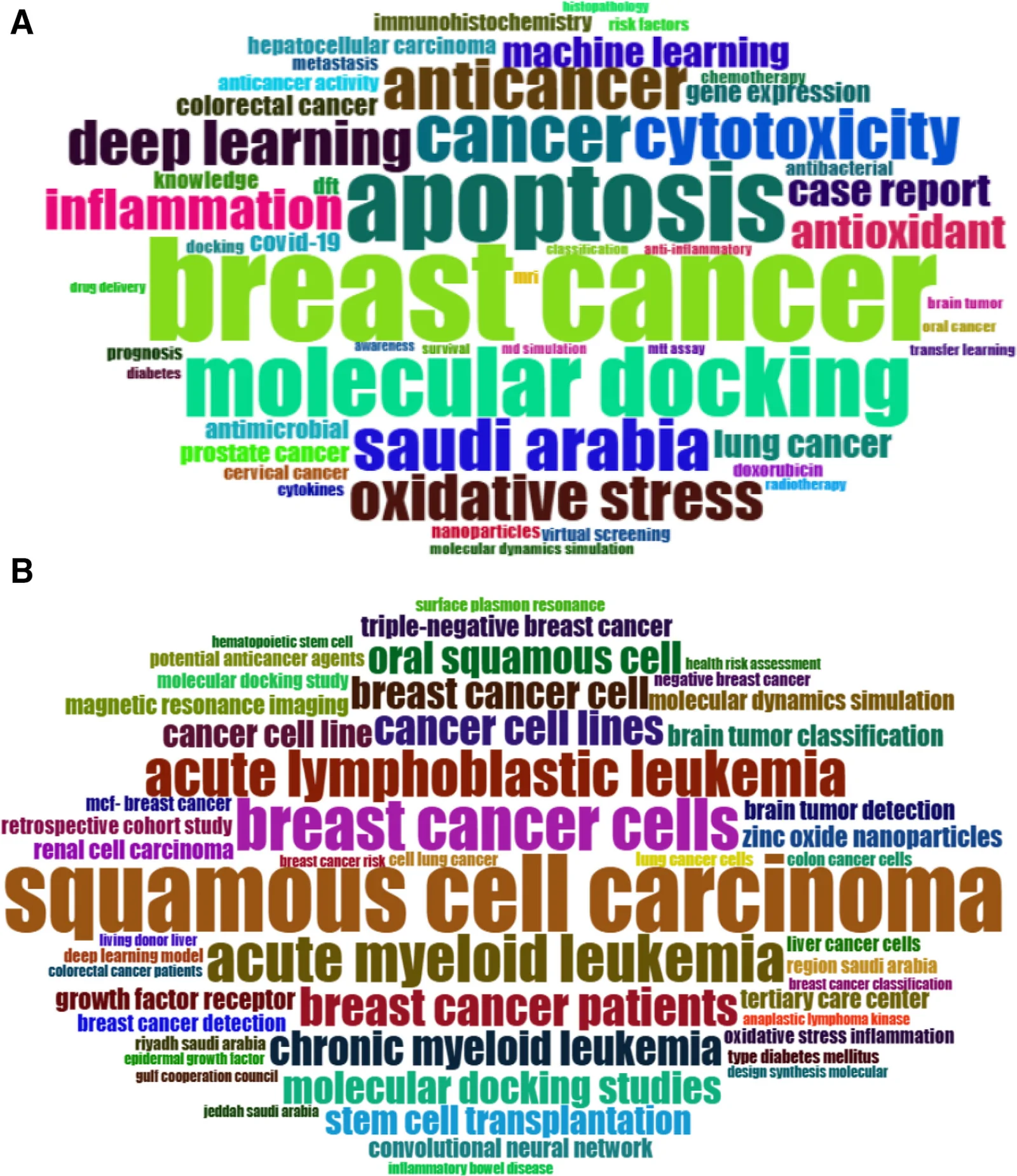
Clustering of terms from author’s keywords (A) and trigrams (three-word combinations) in titles (B).

**Figure 7.**
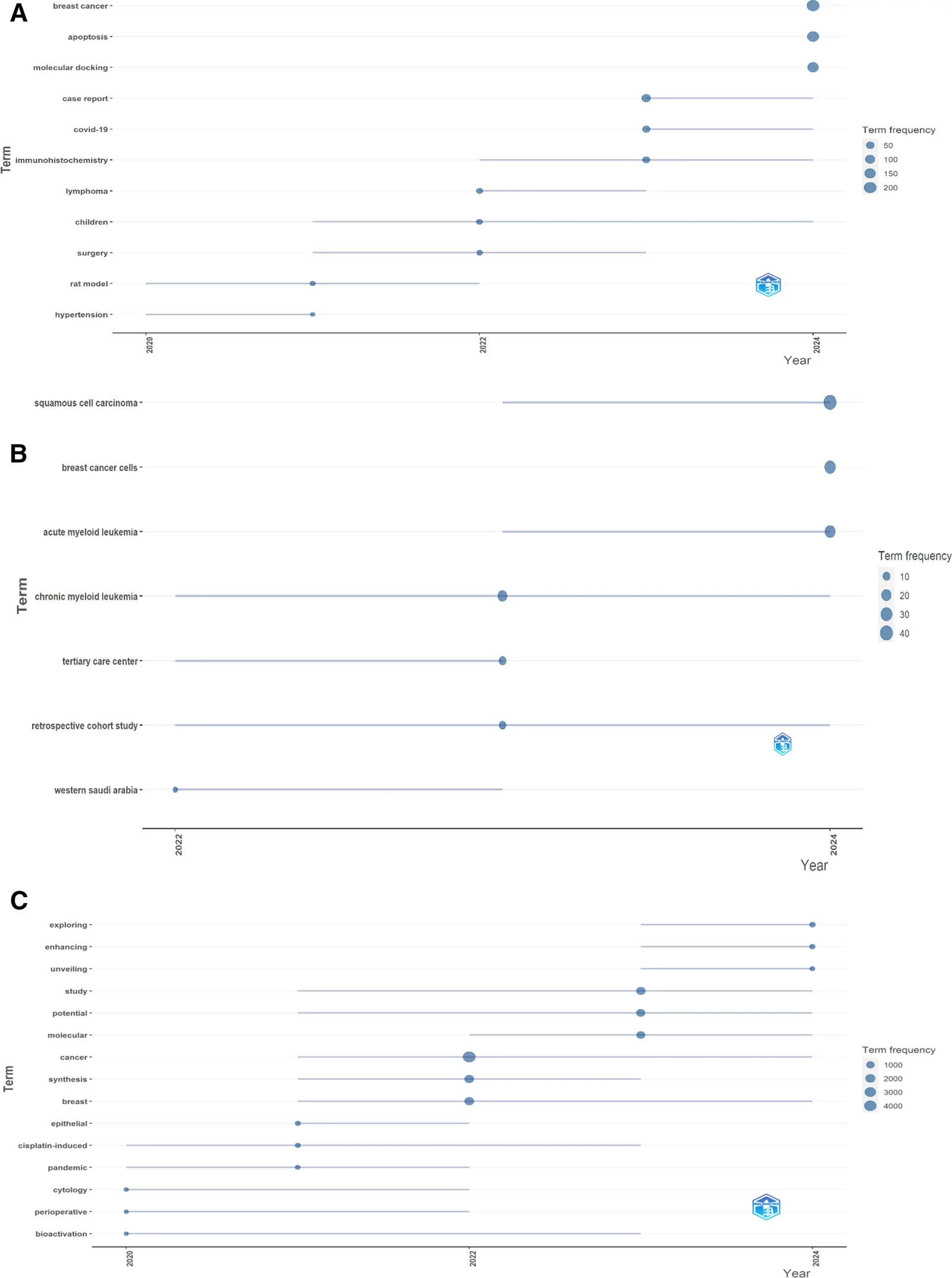
Trending topics analysis in KSA cancer research. (A) Author-provided keywords reveal the most prominent topics, including “breast cancer,” “apoptosis,” “molecular docking,” “COVID-19,” and “lymphoma,” reflecting dominant research areas. (B) Trigram features in titles highlight trending phrases such as “breast cancer cells,” “squamous cell carcinoma,” “acute myeloid leukemia,” and “retrospective cohort study,” emphasizing key themes in clinical and molecular studies. Additionally, (C) unigram analysis of titles extracted frequent terms like “exploring,” “molecular,” “cancer,” and “synthesis,” showcasing a focus on molecular mechanisms, pathways, and advanced techniques. These analyses uncover both established and emerging trends in KSA cancer research. KSA = Kingdom of Saudi Arabia.

## 3. Results

### 3.1. Growth analysis

#### 3.1.1. Growth of cancer research in Kingdom of Saudi Arabia (KSA)

This study highlights the substantial growth in SCR from 1961 to 2024, comprising 40,180 documents (Fig. 2). During the early phase (1961–1999), research output was minimal, with sporadic activity marked by single-digit annual publications, such as one document in 1961, 1970, and 1976, reflecting a limited research infrastructure and focus. The 1980s and the 1990s saw modest progress, with annual outputs gradually increasing but remaining under 0.5% of the total contributions, signaling the infancy of cancer research in the Kingdom. The growth phase (2000–2014) demonstrated a steady rise, starting with 141 publications in 2000 and progressing to 1221 publications in 2014. This period reflects improvements in research capacity, infrastructure, and institutional support, with the research output reaching 3.03% in 2014. The accelerated growth phase (2015–2019) marked a significant turning point, with annual publications consistently exceeding 1000, culminating in 2378 documents in 2019, accounting for 5.91% of the total. This growth indicates a stronger focus on oncology research, increased funding opportunities, and international collaboration. The most remarkable surge occurred in the recent phase (2020–2024), when research productivity reached unprecedented levels. From 3203 publications in 2020 (7.97%), the output soared to 4107 in 2021, 5047 in 2022, 5357 in 2023, and peaked at 5707 in 2024, representing 14.20% of total contributions. Collectively, the period 2020–2024 accounted for over 58% of all cancer research outputs, reflecting a significant national prioritization of oncology research, improved research infrastructure, government funding, and increased collaboration, both nationally and internationally.

The growth of KSA cancer research from 1961 to 2024 follows an exponential regression model (Fig. 2) described by the following equation:


$$Y=1E-123e^{0.1441x}$$

with high goodness of fit (*R*^2^ = 0.9399; *P* < .001). This strong correlation indicates a rapid and consistent increase in cancer research output over time, particularly in recent years, reflecting significant advancements in research capacity, infrastructure, and funding within the Kingdom. This exponential trend highlights Saudi Arabia’s growing commitment to address cancer-related challenges through scientific contributions. Overall, the growth in cancer research since 1961 demonstrates Saudi Arabia’s transformation into a hub for scientific discovery and innovations in oncology. The exponential rise over the last 5 years highlights the country’s commitment to addressing cancer as a major health priority, aligning with national initiatives to improve healthcare outcomes and contribute to global cancer research. These findings underscore the increasing capacity of Saudi Arabia to conduct impactful research, identify emerging trends, and address critical gaps in cancer prevention, diagnosis, and treatment.

#### 3.1.2. Scholarly discourse: comparison between 1961–2024 and 2020–2024

Table 1 provides a comparative analysis of the most productive scholars, affiliations, funders, subjects, and sources of Saudi Arabia’s cancer research for 1961–2024 and 2020–2024. Overall, Patil leads with 298 publications (1961–2024), whereas Aljurf dominates the recent period with 109 publications, reflecting a shift in the leading contributors. King Saud University remains the most productive institution across both periods, contributing 4889 publications overall and 9877 publications in recent years, followed by King Abdulaziz University and rising contributors like Princess Nourah Bint Abdulrahman University. Funders such as King Saud University and international agencies such as the National Institutes of Health play key roles, with notable growth in national funding from 2020 to 2024. Medicine remains the dominant research subject, contributing 19,490 publications overall and 6788 publications in the recent period, alongside increasing contributions in biochemistry, genetics, and molecular biology, Pharmacology, and emerging fields such as Computer Science and Engineering. Leading sources include the *Saudi Medical Journal* (overall leader) and *Molecules* (most prominent in 2020–2024), whereas journals such as *Scientific Reports* and *Annals of Saudi Medicine* continue to play significant roles. These findings highlight the remarkable growth and diversification of cancer research in Saudi Arabia with increased collaboration, funding, and interdisciplinary approaches that have driven recent advancements.

**Table 1:** Scholarly discourse: comparison between 1961–2024 and 2020–2024.

Rank	Scholars				Affiliation					Funders					Subjects				Sources			
	1961–2024		2020–2024		1961–2024		2020–2024			1961–2024			2020–2024		1961–2024		2020–2024		1961–2024		2020–2024	
1	Patil S	298	Aljurf M	109	King Saud University	4889	King Saud University	9877	King Saud University		7231	King Saud University		5000	Medicine	19,490	Medicine	6788	*Saudi Medical Journal*	742	*Molecules*	510
2	Aljurf M	297	Elkaeed EB	88	King Abdulaziz University	2292	King Abdulaziz University	5377	King Abdulaziz University		1244	Princess Nourah Bint Abdulrahman University		915	Biochemistry, genetics and molecular biology	12,671	Biochemistry, genetics and molecular biology	5832	*Molecules*	720	*Scientific Reports*	331
3	Kamal MA	233	Alfaifi MY	85	College of Sciences	2172	King Faisal Specialist Hospital & Research Centre	3968	National Institutes of Health, USA		1146	King Abdulaziz University		812	Pharmacology, toxicology and pharmaceutics	7285	Pharmacology, toxicology and pharmaceutics	3533	*Annals of Saudi Medicine*	498	*Pharmaceuticals*	257
4	Haque S	144	Patil S	84	Princess Nourah Bint Abdulrahman University	1457	College of Sciences	3468	Princess Nourah Bint Abdulrahman University		982	Taif University		690	Chemistry	5886	Chemistry	3464	*Scientific Reports*	450	*Journal of King Saud University Science*	250
5	Kazmi I	138	Alharbi SA	83	King Khalid University	1426	College of Pharmacy	2652	U.S. Department of Health and Human Services		925	King Khalid University		542	Chemical engineering	2506	Chemical engineering	1695	*Plos One*	355	*International Journal of Molecular Sciences*	201
6	Al-Dayel F	137	Chinnathambi A	81	College of Pharmacy	1386	King Khalid University	2321	King Khalid University		821	National Institutes of Health		472	Materials science	2408	Materials science	1543	*International Journal of Molecular Sciences*	348	*Saudi Journal of Biological Sciences*	193
7	Al-Kuraya KS	136	Eissa IH	78	Taif University	1273	Prince Sattam Bin Abdulaziz University	2027	Taif University		761	Prince Sattam Bin Abdulaziz University		441	Engineering	2365	Computer science	1459	*Asian Pacific Journal of Cancer Prevention*	336	*Journal of Molecular Structure*	192
8	Almalki WH	128	Eldehna WM	74	Prince Sattam Bin Abdulaziz University	1228	Umm Al-Qura University	2023	National Cancer Institute		741	Deanship of Scientific Research, King Faisal University		388	Computer science	2270	Engineering	1387	*Pharmaceuticals*	296	*Journal of Biomolecular Structure and Dynamics*	177
9	Sarode SC	128	Hassan MI	74	Umm Al-Qura University	1164	Taif University	1866	Deanship of Scientific Research, King Faisal University		611	U.S. Department of Health and Human Services		369	Immunology and microbiology	1997	Agricultural and biological sciences	1143	*Saudi Pharmaceutical Journal*	272	*Diagnostics*	162
10	Alhakamy NA	125	Elbehairi SEI	71	King Faisal Specialist Hospital & Research Centre	1090	Cairo University	1790	King Abdulaziz City for Science and Technology		609	Deanship of Scientific Research, King Khalid University		368	Agricultural and biological sciences	1975	Physics and astronomy	969	*Saudi Journal of Biological Sciences*	271	*IEEE Access*	158

#### 3.1.3. Document types and focus on original research (1961–2024)

The analysis of document types published in Saudi Arabia’s cancer research between 1961 and 2024 revealed a strong emphasis on original research, with 80.17% of the publications categorized as articles (32,213). Reviews accounted for 13.32% (5351) of the total, whereas other document types, including book chapters, conference papers, and letters, contributed minimally. This dominance of original research highlights the Kingdom’s commitment to generate new scientific knowledge and advance cancer research. The presence of reviews and editorials reflects efforts to synthesize findings and shape research directions. Overall, this distribution underscores Saudi Arabia’s growing research capacity and focuses on its impactful contributions to oncology.

### 3.2. Study 2 (2020–2024)

#### 3.2.1. Main information about the data (2020–2024)

For the period 2020–2024, the bibliometric analysis focused specifically on original articles (N = 18,415), as these publications represent primary peer-reviewed research that drives scientific progress. Focusing on the original articles allows for a more accurate assessment of research productivity, emerging trends, and advancements, thereby reflecting the contribution of Saudi Arabia’s scientific community to global cancer research. Table 2 summarizes the key characteristics of the dataset for the KSA cancer research from 2020 to 2024. A total of 18,154 documents were published across 3253 journals, reflecting the breadth of the research dissemination. The documents have a low average age of 1.76 years, indicating the recency of the data, with an impressive average of 10.85 citations per document, suggesting substantial research impact. The dataset included 68,008 Keywords Plus (ID) and 36,272 author-provided keywords (DE), reflecting diverse and evolving research themes. The analysis included 78,863 authors, with 809 single-author documents representing 643 individual authors. Collaboration was notably high, with an average of 10.6 coauthors per document, underscoring strong teamwork within the research community. Furthermore, 77.12% of the documents resulted from international coauthorships, highlighting the significant global collaboration in advancing cancer research in Saudi Arabia. These findings demonstrate both the productivity and international reach of the KSA’s cancer research efforts during this period.

**Table 2:** Main information about data (2020–2024).

Timespan	2020–2025
Sources (journals)	3253
Documents	18,154
Document average age	1.76
Average citations per document	10.85
Keywords Plus (ID)	68,008
Author’s keywords (DE)	36,272
Authors	78,863
Authors of single-authored documents	643
Authors collaboration	
Single-authored documents	809
Coauthors per document	10.6
International coauthorships (%)	77.12

#### 3.2.2. Global and national landscape of KSA’s cancer research collaboration

The KSA’s cancer research demonstrates extensive global collaboration involving partnerships with 124 countries. As shown in Figure 3A, the top collaborating countries include Egypt, with the highest number of joint publications (2433), followed by India (1627), and the United States (942). Other significant collaborators include Pakistan (862), the United Kingdom (521), China (441), and Canada (274). Additionally, regional neighbors such as Iraq (255), Jordan (231), and the United Arab Emirates (201) feature prominently. These collaborations highlight the strong regional and international partnerships driving KSA’s cancer research, fostering knowledge exchange and advancing scientific progress on a global scale. Figure 3B highlights the ratios of single-country publications and multiple-country publications in the KSA cancer research. An single-country publications ratio of 0.64 indicates that 64% of the KSA’s cancer research output is based on national collaboration, reflecting strong internal research efforts. In contrast, an multiple-country publications ratio of 0.36 indicates that 36% of publications involve international collaborations. This balance underscores the Kingdom’s commitment to fostering local research capacity while maintaining active global partnerships, contributing to knowledge sharing and advancements in cancer research.

Figure 4 illustrates the progression of research article production over time for Saudi Arabia and its top collaborating countries, including Egypt, India, Pakistan, and the United States. Notably, Saudi Arabia showed an increase from 291 articles in 2020 to 8624 articles in 2024, reflecting a significant surge in cancer research productivity. Egypt experienced substantial growth, particularly in 2024 with 2433 articles, following consistent annual increases. Similarly, India’s output will escalate from 19 articles in 2020 to 1627 in 2024 and Pakistan’s output will grow steadily, reaching 862 articles in 2024. The United States demonstrated a more gradual increase, culminating in 942 articles by 2024. These trends highlight Saudi Arabia’s leadership in research production, while emphasizing its collaborative partnerships with key countries driving global cancer research efforts.

#### 3.2.3. Mapping of author’s and institution’s collaboration

The author’s collaboration network in KSA cancer research was mapped, revealing 10 distinct clusters based on betweenness centrality values and visualized with nodes representing individual authors, where the node size indicates the strength of collaboration and colors distinguish clusters (Fig. 5A). Cluster 1 was anchored by Ahmad (64.59), with strong contributions from Khan (64.53), Khan (60.68), and Khan (59.62), highlighting its central role. Cluster 2 was led by Alharbi (78.48) and Alanazi (67.70), while Cluster 3 was dominated by Alfaifi et al (21.29) and El-Kott AF (16.20). Clusters 4 and 5 were anchored by Abdel-Daim (19.02) and Eldehna (29.95), respectively, along with other notable contributors. In Cluster 6, Wang (49.75) and Zhang (29.80) led collaborations, while smaller clusters, such as Cluster 7 (Alharbi SA), Cluster 9 (Chinnathambi A), and Cluster 10 (Alkahtani S) demonstrated limited connectivity. Cluster 8, led by Elkaeed, exhibits minimal collaborative strength. These results highlight the structured and diverse collaboration network, with Cluster 1 serving as the most central group driving KSA cancer research (Fig. 5).

In addition, institutional collaboration networks (Fig. 5B) were analyzed to identify 3 major clusters. Cluster 1 included internationally recognized institutions such as the University of Toronto (5.42), Harvard Medical School (5.40), and the University of Cambridge (4.86), with King Faisal Specialist Hospital and Research Centre playing prominent roles (4.28). Cluster 2 was anchored by local institutions, such as King Saud Bin Abdulaziz University for Health Sciences (1.58) and King Faisal Specialist Hospital and Research Centre (1.09). In Cluster 3, King Saud University exhibited the highest betweenness value (27.65), indicating its central role in the KSA research network, followed by King Abdulaziz University (10.54), and other national collaborators such as Umm Al-Qura University (12.94) and Cairo University (12.61). These findings underscore the collaborative strength of both authors and institutions, with Cluster 1 driving international partnerships, whereas Cluster 3 highlights the dominance of local institutions in advancing KSA cancer research (Fig. 5B).

#### 3.2.4. Conceptual structure of KSA cancer research (2020–2024)

The conceptual structure of KSA cancer research was analyzed using the most frequent terms based on author-provided keywords and trigram features in the titles. Trigrams (three-word combinations) in titles were analyzed to reveal frequently occurring phrases, highlight key research areas, and forecast emerging trends over time. Similarly, author keywords were assessed to determine dominant and evolving topics as they reflect the research focus. According to author keywords (Fig. 6A), the most frequent terms included “breast cancer” (220), “apoptosis” (172), and “molecular docking” (150), reflecting key research focuses on breast cancer, cellular mechanisms, and molecular modeling. Other notable terms included “cytotoxicity” (107), “oxidative stress” (101), and emerging areas like “deep learning” (96) and “machine learning” (63), indicating the integration of artificial intelligence (AI) in cancer research. In contrast, analysis of trigram features in the titles (Fig. 6B) revealed terms such as “squamous cell carcinoma” (40), “breast cancer cells” (27), and “acute myeloid leukemia” (25), emphasizing research on cancer cell types and leukemia. Other prominent terms include “molecular docking studies” (17) and “stem cell transplantation” (15), reflecting efforts in molecular modeling and therapeutic advancements. These findings highlight the dominant focus on breast cancer and molecular research while showcasing an emerging trend toward computational methods and AI in KSA cancer research.

#### 3.2.5. Thematic map analysis

Thematic map analysis was conducted using the Bibliometrix application, which clusters author-provided keywords into 4 distinct themes. These themes were identified based on centrality (relevance degree) and density (development degree), and categorized as basic, motor, niche, or declining.

1. Theme #1: Basic Theme (“Foundational Cancer Research”). This theme represents the core and foundational topics in cancer research and includes keywords such as breast cancer, cancer, Saudi Arabia, lung cancer, colorectal cancer, gene expression, metastasis, chemotherapy, radiotherapy, survival, risk factors, and screening. These keywords reflect the general and widely studied aspects of cancer research central to this field.
2. Theme #2: Declining Theme (“Oxidative Stress and Inflammation”). Keywords in this cluster, including oxidative stress, inflammation, doxorubicin, cytokines, diabetes, cardiotoxicity, cisplatin, and TNF-α, indicate areas that are less relevant and show a declining trend in recent years.
3. Theme #3: Motor Theme (“Advanced Molecular and Drug Discovery Research”). This theme highlights highly developed and actively evolving research areas such as apoptosis, molecular docking, cytotoxicity, anticancer, antioxidant, nanoparticles, drug delivery, drug discovery, virtual screening, molecular dynamics simulation, silver nanoparticles, and EGFR. These keywords reflect significant advancements in molecular mechanisms, therapeutic targets, and computational approaches in cancer research.
4. Theme #4: Niche Theme (“Artificial Intelligence and Imaging Techniques”). Representing highly specialized but less central topics. This theme includes keywords such as deep learning, machine learning, MRI, brain tumors, transfer learning, AI, and classification. These terms point toward emerging applications of AI and advanced imaging techniques in cancer diagnosis and treatment.

These findings underscore the dynamic nature of KSA cancer research, where basic themes address foundational areas, motor themes drive innovation, and niche themes reflect cutting-edge technologies, while certain areas show signs of decline.

#### 3.2.6. Trending topics analysis in KSA cancer research

Trending topic analysis in bibliometric studies was conducted to identify emerging research themes by examining trigram features in titles and author-provided keywords. Trigrams (three-word combinations) in titles were analyzed to reveal frequently occurring phrases, highlight key research areas, and forecast emerging trends over time. Similarly, author keywords were assessed to determine dominant and evolving topics as they reflect the research focus. By analyzing frequency and temporal changes, established themes and emerging directions in KSA cancer research were uncovered. According to author-provided keywords, the most prominent trending topics included “breast cancer,” “apoptosis,” “molecular docking,” “case report,” “COVID-19,” “lymphoma,” and “immunohistochemistry” (Fig. 7A). In contrast, analysis of trigram features in titles revealed trending phrases such as “breast cancer cells,” “squamous cell carcinoma,” “acute myeloid leukemia,” “western Saudi Arabia,” “chronic myeloid leukemia,” “tertiary care center,” and “retrospective cohort study” (Fig. 7B). Additionally, the unigram trend analysis of titles extracted frequently occurring single terms, including “exploring,” “enhancing,” “unveiling,” “molecular,” “epithelial,” “cancer,” “synthesis,” “breast,” “study,” and “potential.” These terms reflect the growing emphasis on exploring molecular mechanisms, cancer pathways, synthesis studies, and the potential of advanced techniques, particularly for breast and epithelial cancers. These findings emphasize significant advancements in breast cancer research, molecular studies, leukemia, and clinical investigations. The analysis, performed using Bibliometrix, provides valuable insights into the current research landscape in KSA cancer research, identifying both well-established and emerging trends (Fig. 7).

### 3.3. Study 3

#### 3.3.1. Main information about SCR (1916–2024) data

Table 3 provides key details about Saudi Arabia’s independent cancer research output from 1961 to 2024. This dataset includes 9319 documents published across 2319 journals, with an impressive annual growth rate of 11.46%, indicating a consistent expansion in research productivity. The average age of documents is 9.74 years, reflecting the historical depth of the data, while each document has received an average of 11.91 citations, highlighting its scientific impact. The dataset contains 33,523 ID and 16,728 DE, reflecting diverse and evolving research topics. A total of 19,651 authors contributed, with 1694 single-author documents and an average of 4.45 coauthors per document, demonstrating a collaborative research culture. These data exclude all international affiliations, focusing solely on research driven by Saudi institutions and offering a comprehensive view of the country’s independent contributions to cancer research.

**Table 3:** Main information about Saudi Cancer Research (SCR) data.

Timespan	1961–2024
Sources (journals)	2319
Documents	9319
Annual growth rate (%)	11.46
Document average age	9.74
Average citations per document	11.91
Keywords Plus (ID)	33,523
Author’s keywords (DE)	16,728
Authors	19,651
Authors of single-authored documents	1243
Authors collaboration	
Single-authored document	1694
Coauthors per document	4.45

#### 3.3.2. Annual growth

**Figure 8.**
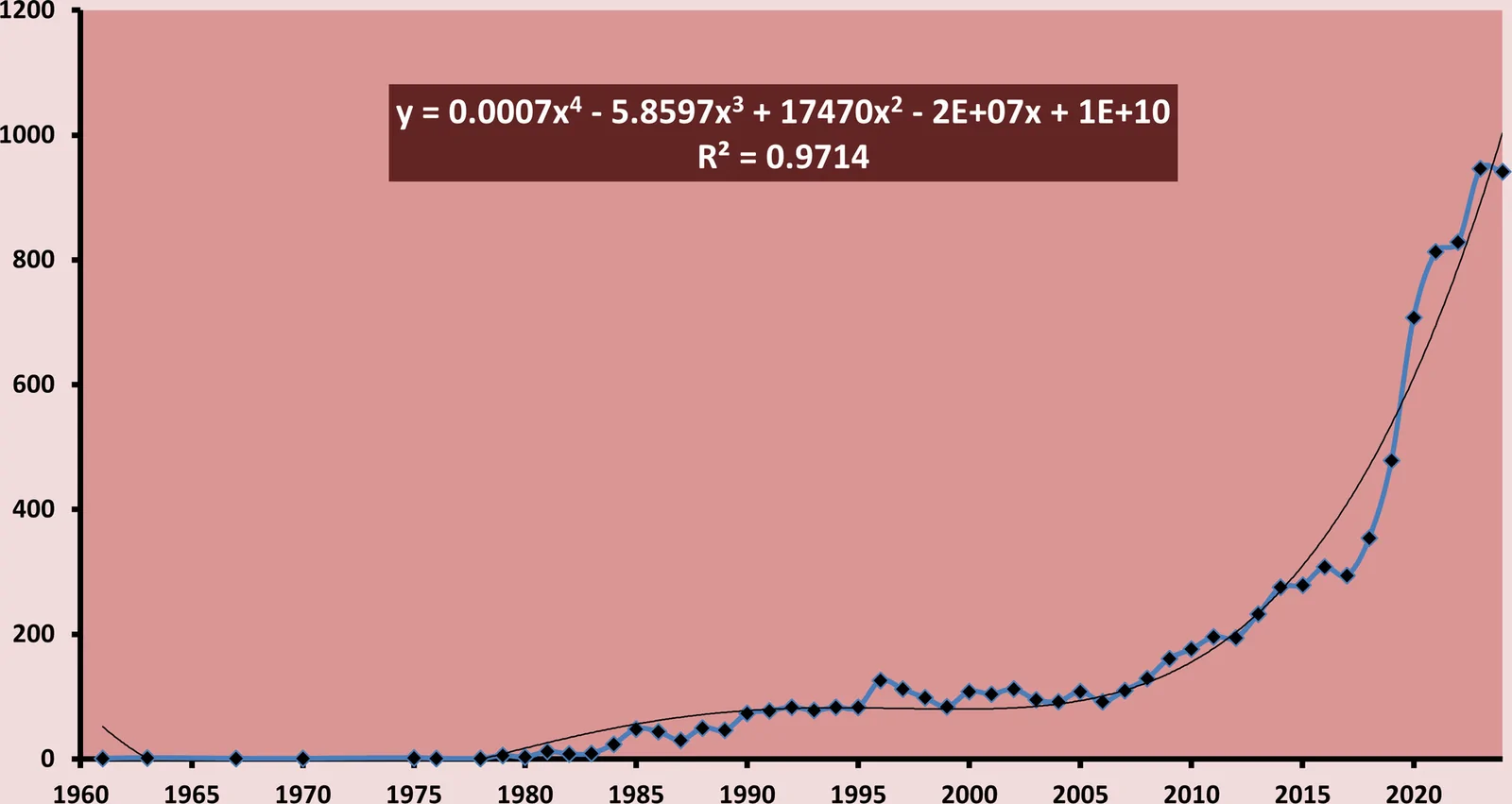
Annual growth for Saudi independent research in cancer.

Figure 8 illustrates the growth of Saudi-specific cancer research from 1961 to 2024, showing the number of published documents per year. A degree 4 polynomial regression was applied to model this growth, achieving a high *R*^2^ value of 0.9714 (*P* < .001), indicating excellent fit and reliability. The equation for the fitted curve is as follows:


$$y=0.0007x^{4}-5.8597x^{3}+17470x^{2}-2\text{E+}07x+1\text{E+}10$$

captures trends in research output over decades. The exponential rise in publications during the last 2 decades has highlighted significant advancements and an increased focus on independent cancer research in Saudi Arabia.

#### 3.3.3. Hotspots

An analysis of independent cancer research data from Saudi Arabia revealed the most productive contributors across various categories. Al-Dayel (118 documents), Al-Kuraya (111 documents), Siraj (88 documents), and Tulbah (60 documents) are the top contributors. The leading affiliations included King Saud University (4600 documents), King Faisal Specialist Hospital and Research Centre (1689 documents), King Abdulaziz University (1016 documents), and King Saud Bin Abdulaziz University for Health Sciences (678 documents). The primary funding entities were King Saud University (730 projects), the Deanship of Scientific Research (661 projects), King Abdulaziz University (292 projects), and King Abdulaziz City for Science and Technology (264 projects). The most prominent sources were *Saudi Medical Journal* (517 publications), *Annals of Saudi Medicine* (382 publications), *International Journal of Surgery Case Reports* (152 publications), and *Saudi Pharmaceutical Journal* (120 publications). The dominant research areas included Medicine (6296 documents); biochemistry, genetics, and molecular biology (1989 documents); pharmacology, toxicology, and pharmaceuticals (1021 documents); and chemistry (656 documents). These results highlight the critical role of leading institutions, funders, and journals in advancing independent cancer research in Saudi Arabia, with strong emphasis on medicine and molecular biology. The additional details are presented in Table 4.

**Table 4:** Hotspots.

Ranks	Author name	N	Affiliation	N	Funders	N	Source title	N	Subject area	N
1	Al-Dayel F	118	King Saud University	4600	King Saud University	730	*Saudi Medical Journal*	517	Medicine	6296
2	Al-Kuraya KS	111	King Faisal Specialist Hospital & Research Centre	1689	Deanship of Scientific Research, King Saud University	661	*Annals of Saudi Medicine*	382	Biochemistry, genetics and molecular biology	1989
3	Siraj AK	88	King Abdulaziz University	1016	King Abdulaziz University	292	*International Journal of Surgery Case Reports*	152	Pharmacology, toxicology and pharmaceutics	1021
4	Tulbah A	60	King Saud Bin Abdulaziz University for Health Sciences	678	King Abdulaziz City for Science and Technology	264	*Saudi Pharmaceutical Journal*	120	Chemistry	656
5	Parvathareddy SK	57	Imam Abdulrahman Bin Faisal University	672	Ministry of Education, Kingdom of Saudi Arabi	122	*American Journal of Case Reports*	112	Chemical engineering	372
6	Aboussekhra A	55	National Guard Health Affairs	440	Prince Sattam Bin Abdulaziz University	119	*Asian Pacific Journal of Cancer Prevention*	111	Agricultural and biological sciences	343
7	Ahamed M	52	King Khalid University Hospital	416	Princess Nourah Bint Abdulrahman University	106	*Saudi Journal of Biological Sciences*	100	Immunology and microbiology	310
8	Al-Maghrabi J	52	King Abdulaziz Medical City, Riyadh	391	National Plan for Science, Technology and Innovation	95	*Molecules*	79	Computer science	298
9	Alzahrani AS	52	Prince Sultan Military Medical City	340	Deanship of Scientific Research, King Faisal University	90	*Journal of King Saud University Science*	78	Materials science	289
10	Ezzat A	51	King Fahad Medical City	316	King Faisal Specialist Hospital & Research Centre	82	*Journal of Surgical Case Reports*	68	Engineering	288

#### 3.3.4. Most impactful entities in Saudi Arabia’s independent cancer research

The analysis of author impact in Saudi Arabia’s independent cancer research highlights the significant contributors, based on their productivity and influence (Table 5). Al-Dayel F leads with an Hirsch Index (*h*-index) of 33, *g*-index of 51, and 3193 citations from 118 publications since 1991, reflecting a long-standing research career. Al-Kuraya KS follows with an *h*-index of 32, *g*-index of 50, and *m*-index of 1.6, achieving 3025 citations across 110 publications since 2005. Uddin S shows a sustained impact with an *h*-index of 31, a *g*-index of 49, and 2409 citations from 51 publications since 2006, while Ahmed and Akhtar M demonstrate long-term contributions with over 2200 and 1623 citations, respectively, from their decades-long careers. Recent researchers such as Ahamed and M. exhibit a high impact with an *m*-index of 2, achieving 2034 citations from 51 publications since 2012, reflecting rapid progress. Other influential authors include Bavi P, Alshatwi AA, Siraj AK, and Tulbah A, with *h*-indices ranging from 24 to 26 and substantial citations. These metrics underscore the diverse contributions of authors across different timeframes, with recent studies showing a growing impact that shapes the future of cancer research in Saudi Arabia.

**Table 5:** Author impact.*

Element	*h*-index	*g*-index	*m*-index	TC	NP	PY_start
Al-Dayel F	33	51	0.971	3193	118	1991
Al-Kuraya KS	32	50	1.60	3025	110	2005
Uddin S	31	49	1.632	2409	51	2006
Ahmed M	30	45	0.811	2200	69	1988
Akhtar M	27	38	0.614	1623	74	1981
Ahamed M	26	44	2.00	2034	51	2012
Bavi P	26	37	1.368	1946	37	2006
Alshatwi AA	25	40	1.667	1660	47	2010
Siraj AK	24	40	0.889	1930	90	1998
Tulbah A	24	49	0.80	2479	60	1995

* The *h*-index measures a researcher’s productivity and impact, representing the number of publications (*h*) with at least *h* citations. The *g*-index gives more weight to highly-cited papers, indicating the top-cited works with at least *g*^2^ citations. The *m*-index normalizes the *h*-index by years since the first publication (PY_start), showing career impact rate. Total citations (TC) reflect cumulative citation influence, while number of publications (NP) highlights productivity. PY_start marks the first publication year, providing context for career progression. These metrics collectively evaluate a researcher’s productivity, impact, and career trajectory.

#### 3.3.5. Conceptual structure of KSA cancer research (2020–2024)

**Figure 9.**
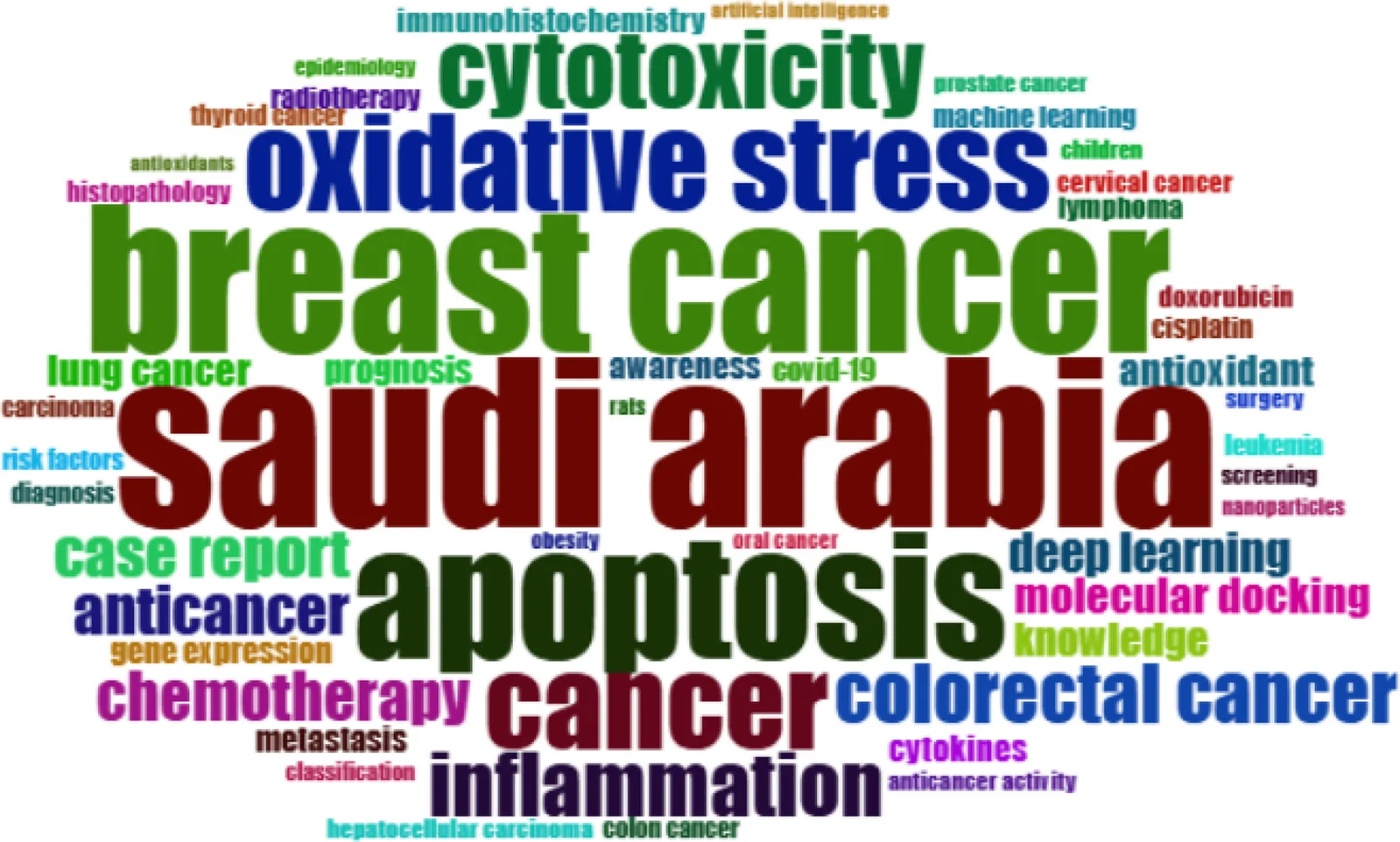
Conceptual structure of KSA cancer research (1961–2024). KSA = Kingdom of Saudi Arabia.

Figure 9 shows the most frequently author-provided keywords in Saudi Arabia’s independent cancer research output from 1961 to 2024, providing insight into the conceptual structure of the field. The top keywords include “Saudi Arabia” (424 mentions), “breast cancer” (373), and “apoptosis” (315), highlighting a focus on regional research, prevalent cancers, and molecular mechanisms. Other frequent terms such as “oxidative stress” (243), “cytotoxicity” (198), “inflammation” (162), and “colorectal cancer” (152) reflect key areas of interest and investigation. Emerging trends are evident from keywords such as “deep learning” (97) and “molecular docking” (91), emphasizing advanced computational and molecular techniques, while terms such as “chemotherapy” (123) and “anticancer” (121) demonstrate a continued focus on therapeutic strategies. These keywords collectively outline the evolving priorities and advancements in independent cancer research in Saudi Arabia.

#### 3.3.6. Thematic mapping

Thematic map analysis, conducted using the Bibliometrix application, identified 4 distinct research themes based on clustering of author-provided keywords. Themes were categorized according to their centrality (relevance) and density (development), classifying them as motor, basic, niche, or declining.

1. Theme #1: Motor Theme (“Comprehensive Cancer Research”) encompasses highly relevant and well-developed topics that drive cancer research in Saudi Arabia. Keywords: Saudi Arabia; breast cancer; cancer; colorectal cancer; case report; chemotherapy; knowledge; lung cancer; prognosis; awareness; metastasis; immunohistochemistry; COVID-19; lymphoma; radiotherapy; cervical cancer; histopathology; risk factors; thyroid cancer; carcinoma; diagnosis; leukemia; hepatocellular carcinoma; surgery; children; prostate cancer.
2. Theme #2: Declining Theme (“Oxidative Stress and Inflammation”) which represents research areas with reduced activity or focus. Keywords: oxidative stress; inflammation; cytokine; gene expression; doxorubicin; colon cancer.
3. Theme #3: Basic Theme (“Molecular and Anticancer Mechanisms”). This theme reflects foundational and widely studied topics in cancer research. Keywords: apoptosis; cytotoxicity; anticancer; molecular docking; antioxidant; cisplatin; anticancer activities.
4. Theme #4: Niche Theme (“Artificial Intelligence in Cancer Research”). This theme covers highly specialized and emerging areas with limited centrality, but high development. The keywords used included deep learning and machine learning.

These results provide insights into the current priorities and evolution of cancer research in Saudi Arabia, with a focus on comprehensive cancer studies, foundational molecular mechanisms, and emerging AI-driven approaches, although some areas have shown declining interest.

#### 3.3.7. Thematic evolution

**Figure 10.**
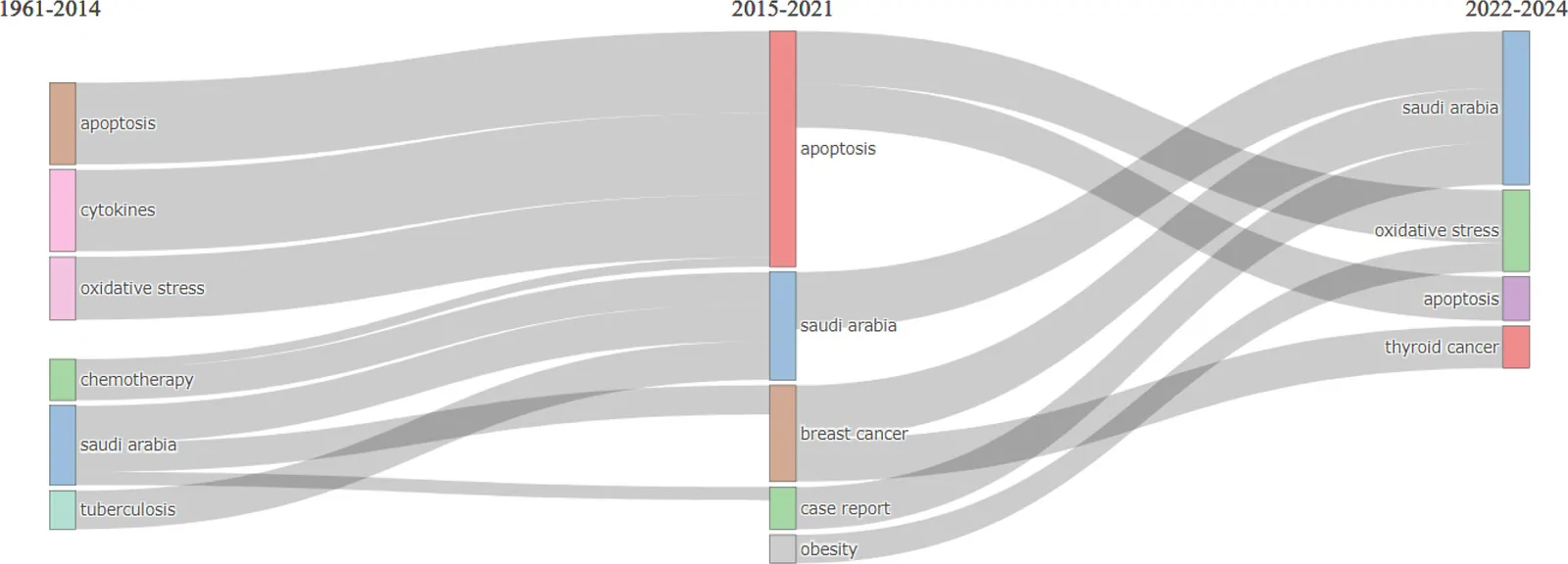
Thematic evolution (1961–2024).

Figure 10 illustrates the thematic evolution of Saudi Arabia’s independent cancer research output from 1961 to 2024, demonstrating the progression and transformation of research priorities over time. Early themes (1961–2014) such as “chemotherapy,” “cytokines,” and “oxidative stress” transitioned into more specific focuses during 2015–2021, including “apoptosis,” “breast cancer,” and “case reports.” In the most recent period (2022–2024), themes have further evolved to incorporate cutting-edge topics like “deep learning,” “thyroid cancer,” and the continued emphasis on “apoptosis” and “oxidative stress.” This progression highlights the shift from broad foundational topics to more specialized and technology-driven areas, reflecting advancements in research methodologies and the integration of modern tools in Saudi Arabia’s cancer research landscape.

#### 3.3.8. Trending topics in Saudi Arabia’s independent cancer research

**Figure 11.**
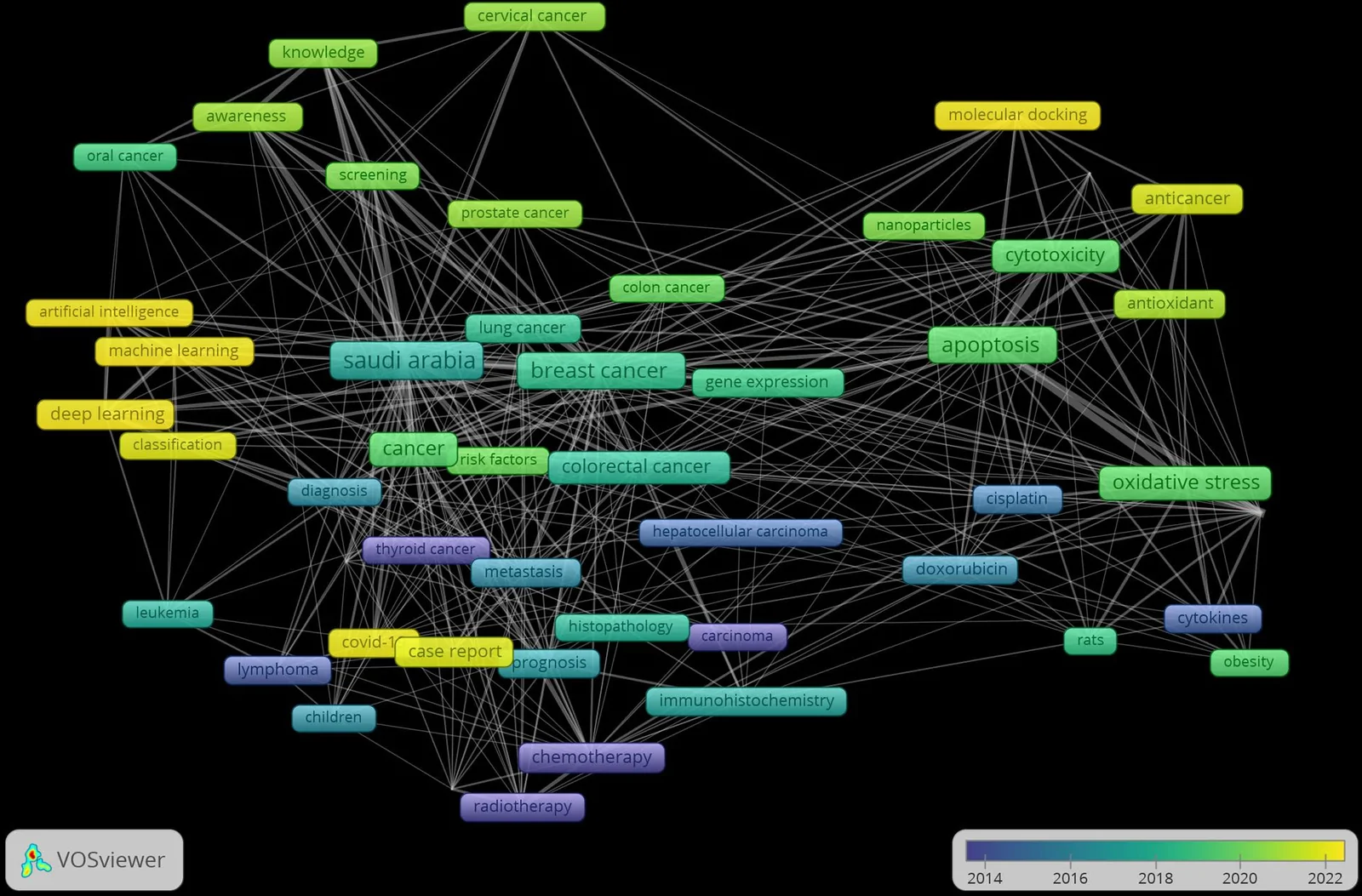
Trending topics in Saudi Arabia’s cancer research (1961–2024). This overlay visualization was created using VOSviewer to map the most frequent author-provided keywords with a minimum of 40 occurrences. The figure highlights the evolution of research focus over time, where yellow nodes indicate trending and emerging topics. These nodes represent areas of recent research interest, reflecting the dynamic and evolving priorities in Saudi Cancer Research. This visualization offers valuable insights into the current and future directions of the research landscape.

Using VOSviewer, overlay visualization was used to show the changes in research priorities through the mapping of the most frequently used author-provided keywords with a minimum frequency of 40, as shown in Figure 11. The findings indicated yellow nodes in the visualization, which are emerging and trending topics within the field of independent cancer research in Saudi Arabia. These topics include molecular docking, anticancer, deep learning, AI, classification, case reports, and COVID-19. The findings also demonstrate an increasing concentration of the use of complex computational methods, molecular research, and solutions to healthcare problems from practice, demonstrating the active and progressive state of research in the country.

### 3.4. Study 4

The fourth part of this study focused exclusively on original research studies (N = 4240) conducted solely within Saudi institutions, without any international collaboration, over the past 5 years (2020–2024).

#### 3.4.1. Main information about SCR (2020–2024) data

Table 6 provides an overview of the main characteristics of SCR data from 2020 to 2024. During this period, 4240 documents were published in 1229 journals, reflecting an annual growth rate of 7.56%. The documents had an average age of 1.86 years and received an average of 5.722 citations per document. The dataset includes 22,791 ID and 10,694 DE, showing a rich conceptual structure. The data reflect contributions from 11,473 authors, with 738 single-authored documents, and an average of 5.02 coauthors per document, emphasizing significant collaboration among researchers. These metrics highlight the dynamic and evolving nature of SCR during this period.

**Table 6:** Main information about Saudi Cancer Research (2020–2024) data.

Timespan	2020–2024
Sources (journals)	1229
Documents	4198
Annual growth rate (%)	7.56
Document average age	1.86
Average citations per document	5.722
Keywords Plus (ID)	22,791
Author’s keywords (DE)	10,694
Authors	11,473
Authors of single-authored docs	588
Authors collaboration	
Single-authored document	738
Coauthors per document	5.02

#### 3.4.2. Annual growth

**Figure 12.**
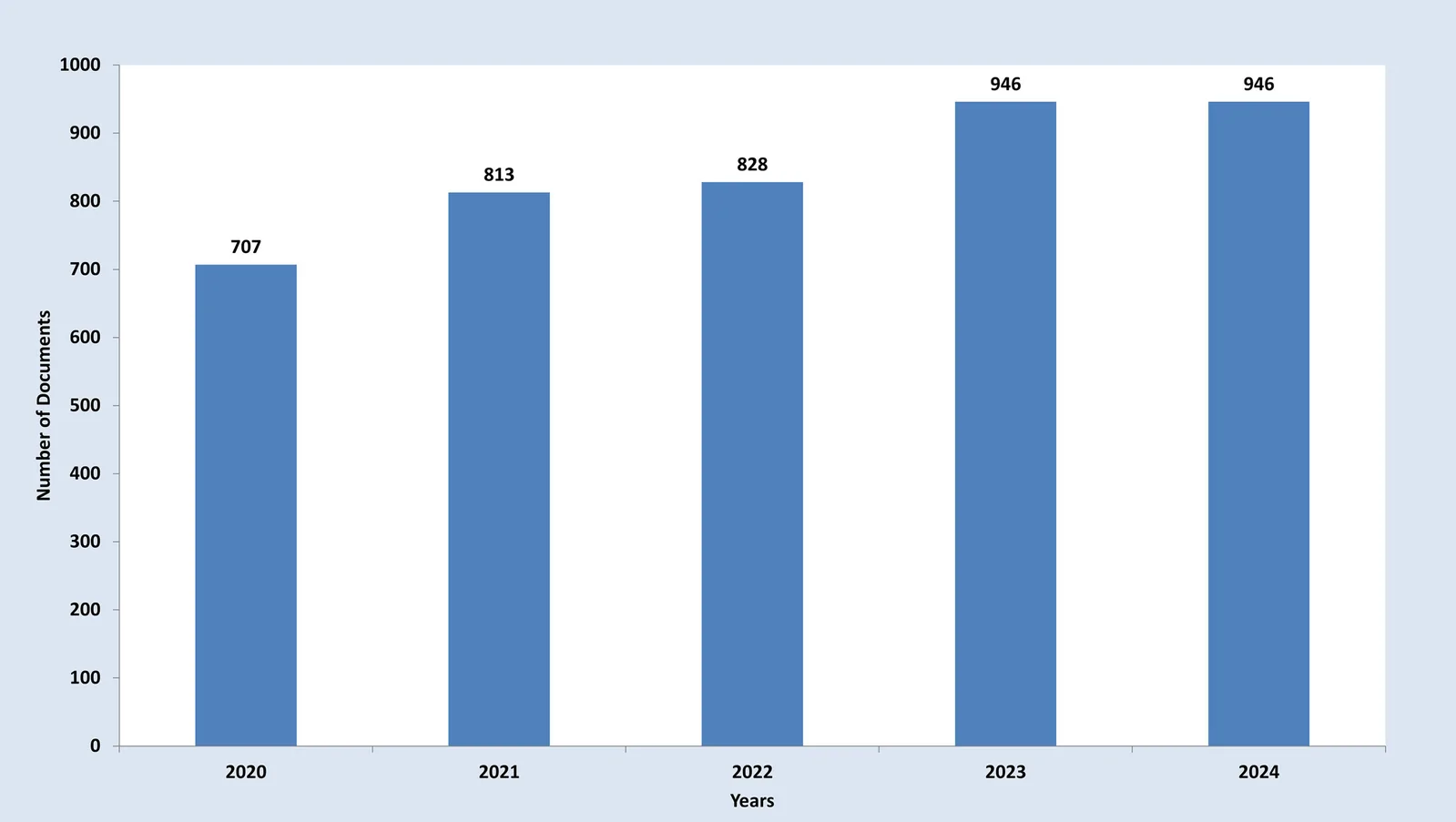
Annual production for the Saudi Arabia’s independent cancer research during the period 2020–2024.

Figure 12 illustrates the annual contribution to Saudi Arabia’s independent cancer research from 2020 to 2024. A total of 4198 documents were published, with the highest contributions recorded in 2023 and 2024 (946 documents, constituting 22.31% of the total for each year). The research output showed consistent growth, with 828 documents (19.53%) in 2022, 813 (19.17%) in 2021, and 707 (16.67%) in 2020. This progress highlights the sustained and increasing emphasis on cancer research in Saudi Arabia.

#### 3.4.3. Hotspots in Saudi Arabia’s independent cancer research (2020–2024)

The analysis of Saudi Arabia’s independent cancer research from 2020 to 2024, excluding global collaboration, identified key contributors, institutions, funders, journals, and subject areas that shaped the research landscape (Table 7). The top affiliations included King Saud University (2406 publications), King Abdulaziz University (574), and the King Faisal Specialist Hospital and Research Centre (453). The key funders were King Saud University (571 projects) and the Deanship of Scientific Research at King Saud University (484 projects). The most prolific journals were the *International Journal of Surgery Case Reports* (106 articles) and *American Journal of Case Reports* (91). Medicine dominated this subject area with 2263 publications, followed by biochemistry, genetics, and molecular biology (994). Among the most productive authors (Table 8), Siraj (49 publications) led the rankings, followed by Al-Kuraya (47), Parvathareddy (46), Al-Dayel (45), and Ahmad (36). This highlights Saudi Arabia’s focus on medicine, biochemistry, and pharmacology, with significant institutional and financial support driving research productivity.

**Table 7:** Hotspots (2020–2024).

Ranks	Author name	N	Affiliation	N	Funders	N	Source title	N	Subject area	N
1	Siraj AK	49	King Saud University	2406	King Saud University	571	*International Journal of Surgery Case Reports*	106	Medicine	2263
2	Al-Kuraya KS	47	King Abdulaziz University	574	Deanship of Scientific Research, King Saud University	484	*American Journal of Case Reports*	91	Biochemistry, genetics and molecular biology	994
3	Parvathareddy SK	46	King Faisal Specialist Hospital & Research Centre	453	King Abdulaziz University	221	*Saudi Pharmaceutical Journal*	80	Pharmacology, toxicology and pharmaceutics	592
4	Al-Dayel F	45	King Saud Bin Abdulaziz University for Health Sciences	446	Ministry of Education, Kingdom of Saudi Arabi	118	*Saudi Medical Journal*	78	Chemistry	445
5	Ahmad SF	36	National Guard Health Affairs	324	Prince Sattam Bin Abdulaziz University	112	*Journal of King Saud University Science*	77	Chemical engineering	301
6	Alshammari GM	34	Imam Abdulrahman Bin Faisal University	267	King Abdulaziz City for Science and Technology	105	*Saudi Journal of Biological Sciences*	73	Computer science	253
7	Nadeem A	33	Princess Nourah Bint Abdulrahman University	226	Princess Nourah Bint Abdulrahman University	98	*Molecules*	68	Agricultural and biological sciences	231
8	Attia SM	32	King Abdulaziz Medical City, Riyadh	223	Deanship of Scientific Research, Prince Sattam Bin Abdulaziz University	68	*International Journal of Molecular Sciences*	56	Materials science	210
9	Ansari MA	31	Prince Sattam Bin Abdulaziz University	197	Taif University	63	*Journal of Surgical Case Reports*	54	Engineering	204
10	Al-Sobhi SS	30	Umm Al-Qura University	194	King Khalid University	53	*Pharmaceuticals*	47	Multidisciplinary	190

**Table 8:** Author impact.*

Element	*h*-index	*g*-index	*m*-index	TC	NP	PY_start
Ahmad SF	13	21	2.6	492	36	2020
Ahamed M	12	23	2.4	567	28	2020
Akhtar MJ	12	21	2.4	466	21	2020
Ansari MA	12	23	2.4	559	37	2020
Nadeem A	12	20	2.4	463	33	2020
Ahmad A	11	20	2.2	413	31	2020
Al-Dayel F	11	18	2.2	407	45	2020
Al-Harbi NO	11	15	2.2	331	15	2020
Al-Kuraya KS	11	18	2.2	422	47	2020
Alanazi MM	11	17	2.2	336	31	2020

* The *h*-index measures a researcher’s productivity and impact, representing the number of publications (*h*) with at least *h* citations. The *g*-index gives more weight to highly-cited papers, indicating the top-cited works with at least *g*^2^ citations. The *m*-index normalizes the *h*-index by years since the first publication (PY_start), showing career impact rate. Total citations (TC) reflect cumulative citation influence, while number of publications (NP) highlights productivity. PY_start marks the first publication year, providing context for career progression. These metrics collectively evaluate a researcher’s productivity, impact, and career trajectory.

#### 3.4.5. Conceptual structure of KSA cancer research (2020–2024)

**Figure 13.**
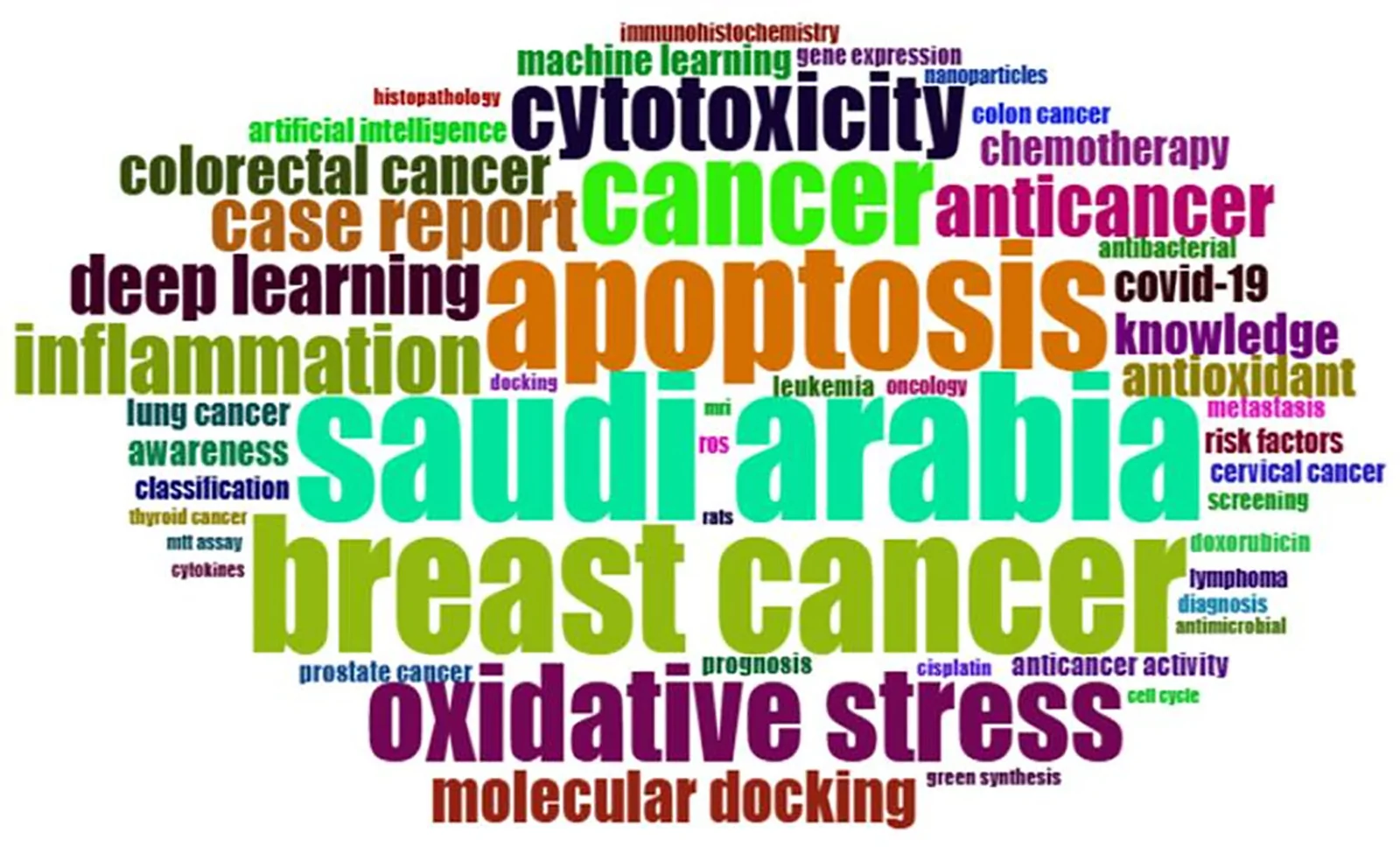
Conceptual structure of KSA cancer research (2020–2024). KSA = Kingdom of Saudi Arabia.

The fourth part of this study examined 4240 original research studies conducted solely within Saudi institutions, without international collaboration, over the past 5 years (2020–2024). By analyzing author-provided keywords, the conceptual structure of SCR during this period was mapped (Fig. 13). The most frequently occurring terms include “Saudi Arabia” (234 occurrences), “breast cancer” (216), “apoptosis” (202), “cancer” (162), and “oxidative stress” (153), reflecting the focus on foundational and emerging research themes. Other notable terms such as “cytotoxicity” (124), “inflammation” (112), “deep learning” (95), and “molecular docking” (83) highlight advancements in molecular research and AI applications in cancer studies.

#### 3.4.6. The thematic map analysis

Thematic map analysis, conducted using the Bibliometrix application, classifies Saudi Arabia’s independent cancer research (2020–2024) into 4 distinct themes based on the clustering of author-provided keywords. These themes were identified according to their centrality (relevance) and density (development) and categorized as motor, basic, emerging, or niche.

1. Theme #1: Motor Theme (“Comprehensive Cancer Research”). This theme represents a highly relevant and well-developed topic in SCR, encompassing keywords such as Saudi Arabia; breast cancer; cancer; colorectal cancer; knowledge; chemotherapy; awareness; lung cancer; risk factors; metastasis; immunohistochemistry; thyroid cancer; recurrence; survival; HPV; and quality of life. These topics address general and widely studied areas, and form the backbone of SCR.
2. Theme #2: Basic Theme (“Artificial Intelligence in Cancer Research”). This includes foundational and steadily developing areas in cancer research, focusing on advancements in AI applications. Keywords: deep learning; machine learning; artificial intelligence; classification; MRI; magnetic resonance imaging; transfer learning.
3. Theme #3: Emerging Theme (“Molecular and Therapeutic Innovations”). This theme highlights rapidly evolving research areas with keywords such as apoptosis; cytotoxicity; anticancer; molecular docking; nanoparticles; green synthesis; drug delivery; anti-inflammatory; and silver nanoparticles. These represent innovations in molecular mechanisms, drug development, and targeted therapies.
4. Theme #4: Niche Theme (“Oxidative Stress and Chronic Conditions”). This theme covered specialized but less central areas, including keywords such as oxidative stress; inflammation; cisplatin; cytokines; antioxidants; diabetes; obesity; and nephrotoxicity. These studies have focused on the interplay between oxidative stress and chronic conditions in cancer research.

This analysis provides valuable insights into Saudi Arabia’s cancer research, identifying strengths in comprehensive and foundational studies, while highlighting emerging innovations and niche explorations that complement the broader research landscape.

#### 3.4.7. Thematic evolution

**Figure 14.**
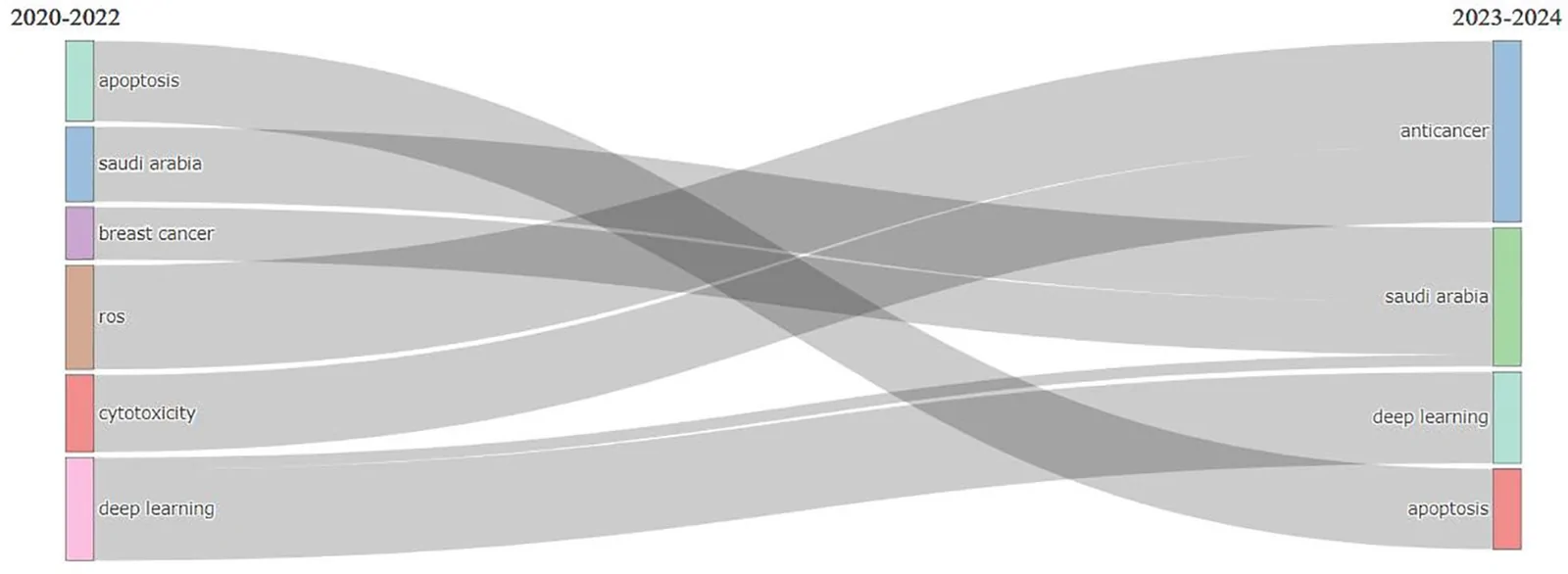
Thematic evolution (2020–2024).

The thematic evolution of SCR within Saudi affiliations from 2020 to 2024 is depicted in Figure 14. This highlights the progress and interconnection of key research themes over time. For instance, “apoptosis” (2020–2022) has evolved into “apoptosis” (2023–2024) and expanded its focus to themes like “Saudi Arabia” (2023–2024). Similarly, “breast cancer” (2020–2022) transitioned into diverse emerging themes, including “anticancer” (2023–2024), “deep learning” (2023–2024), and “Saudi Arabia” (2023–2024), reflecting the integration of AI into cancer research. Topics such as “cytotoxicity” (2020–2022) and “ros” (2020–2022) also converged into the “anticancer” (2023–2024) theme, indicating advancements in therapeutic research. This analysis underscores the dynamic evolution and increasing alignment of SCR using cutting-edge technologies and therapeutic strategies.

#### 3.4.8. Trending topics in Saudi Arabia’s independent cancer research (2020–2024)

**Figure 15.**
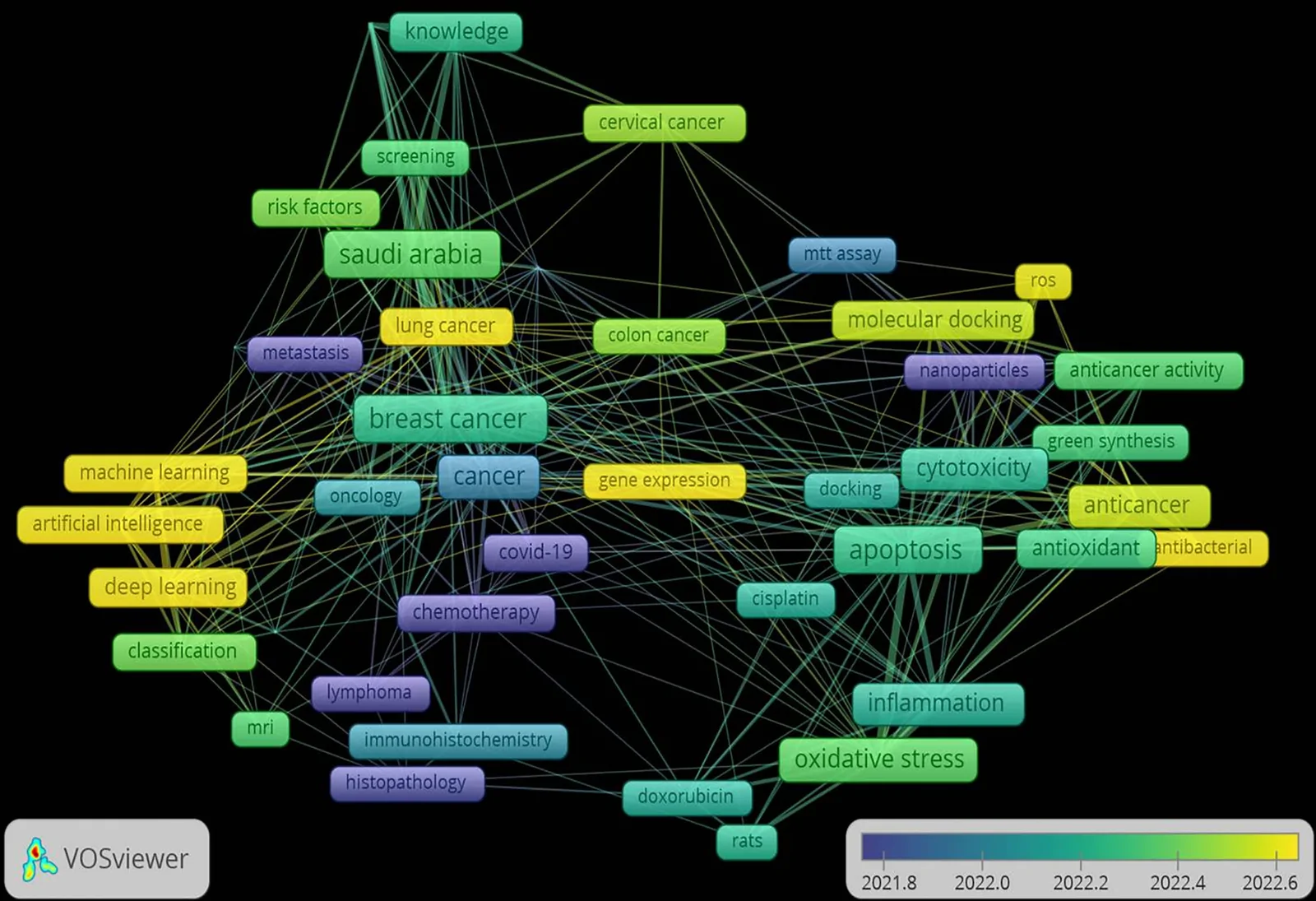
Trending topics in Saudi Arabia’s cancer research (2020–2024). This overlay visualization was created using VOSviewer to map the most frequent author-provided keywords with a minimum of 30 occurrences. The figure highlights the evolution of research focus over time, where yellow nodes indicate trending and emerging topics. These nodes represent areas of recent research interest, reflecting the dynamic and evolving priorities in Saudi Cancer Research. This visualization offers valuable insights into the current and future directions of the research landscape.

Overlay visualization, generated using VOSviewer, mapped the most frequent author-provided keywords with a minimum threshold of 30 occurrences, showcasing the evolution of research priorities over time in Saudi Arabia’s independent cancer research (2020–2024), without global collaboration (Fig. 15). The analysis highlights yellow nodes representing trends and emerging topics in the field. These include gene expression, deep learning, AI, machine learning, reactive oxygen species, lung cancer, molecular docking, and cervical cancer, reflecting the dynamic and innovative areas that currently shape the research landscape.

## 4. Discussion

Thus, the purpose of this study was to evaluate the cancer research output of Saudi Arabia, as the primary aim was to provide ideas and guidance for future national projects. Considering the increase in cancer burden in the Kingdom, the first part of this study assessed global involvement in SCR. Doing so laid out the work done in collaboration with foreign institutions, and sought major focal points. The third part focused exclusively on independent Saudi research, assessing publications solely from Saudi institutions to uncover national productivity, emerging trends, and gaps in addressing local cancer challenges. The fourth part of this study focused exclusively on original research studies (N = 4240) conducted solely within Saudi institutions, without any international collaboration, over the past 5 years (2020–2024). Regardless of the measures in terms of prevention, diagnosis, and treatment, the rising trend in cancer cases and deaths in Saudi Arabia highlights the gap that needs to be addressed in terms of improved research coverage. This study utilized bibliometric analysis, which has become common in geographic cancer research in different parts of the world and the Arab region, to investigate historical trends in SCR. This analysis examined scientific productivity originating from Saudi universities and other institutions, including clinical trials, and observational and epidemiological research.

Since 1961, the advancement of cancer research has illustrated Saudi Arabia’s evolution as a center for scientific discovery and innovation in oncology. Within the last 5 years, a particularly significant growth was noted, depicting the country’s firm resolution to allocate resources towards more pressing health issues, namely cancer, in alignment with international endeavors aimed at improving healthcare delivery systems and advancing cancer treatment worldwide. To interpret this remarkable growth in cancer research productivity from 2020 to 2024, it is important to acknowledge the possible influence of the increasing national cancer burden. Although this bibliometric study did not directly analyze epidemiological data, several reports have highlighted the rising incidence of various cancers in the Kingdom, including breast, colorectal, thyroid, and prostate cancer. This escalating burden likely prompted a heightened governmental and institutional focus on cancer control, contributing to enhanced funding, infrastructure development, and research incentives. Therefore, the observed surge in research output may partially be attributed to a strategic response to the country’s growing healthcare needs in oncology. These results also demonstrate that Saudi Arabia is increasingly positioned to conduct quality research, identify particular waves of change, and meet critical gaps in the cancer prevention, detection, and treatment continuum. The observed findings align with prior bibliometric investigations in regions such as the KSA,^[6]^ UAE,^[21]^ and Arab countries.^[22]^

Dr Mahmoud Deeb Aljurf, affiliated with the King Faisal Specialist Hospital & Research Centre, Riyadh, Saudi Arabia, is the most prolific scholar in SCR in the second study (2020–2024) as shown in Table 1. He has published 477 documents with 18,116 citations across 13,664 documents, achieving a remarkable *h*-index of 72, reflecting both the quantity and impact of his work. Dr Aljurf’s highly cited research primarily focused on acute myeloid leukemia (AML), hematopoietic cell transplantation, and COVID-19 outcomes in patients with cancer. His notable works include updates on the molecular pathogenesis and therapeutic targeting of AML,^[23]^ systematic reviews on transplantation and prognosis in leukemia patients,^[24]^ and COVID-19 infection risk in cancer patients.^[25]^ Other key contributions include studies on transplant-associated thrombotic microangiopathy^[26]^ and relapse-free survival in patients undergoing hematopoietic cell transplantation.^[27]^ His latest research (in 2024) will continue to contribute significantly to this field. Recent studies include trends in allogeneic transplantation for favorable-risk AML,^[28]^ feature extraction frameworks for AML detection,^[29]^ evidence-based guidelines for severe aplastic anemia treatment,^[30]^ longitudinal studies on transplantation trends,^[31]^ and autologous stem cell transplantation in lymphoma.^[32]^ This extensive body of work highlights Dr Aljurf’s critical contributions to leukemia research, advanced therapies, and clinical outcomes, thereby solidifying his status as a leader in SCR.

In the third part of this study, Al-Dayel and Al-Kuraya, both affiliated with King Faisal Specialist Hospital and Research Centre, Saudi Arabia, were identified as the most prolific scholars. Working in the same research group, they have contributed significantly to the independent cancer research landscape in Saudi Arabia. Their notable works include studies on KRAS isoforms and mutations in colorectal carcinoma^[33]^ and clinicopathological analysis of CRCs with PIK3CA mutations in Middle Eastern populations.^[34]^ They also explored PIK3CA alterations in papillary thyroid cancer^[35]^ and the association of FoxM1 with matrix metalloproteinases in papillary thyroid carcinoma.^[36]^ Other important studies include their work on standardizing definitions of hematopoietic recovery in allogeneic stem cell transplantation^[37]^ and the role of bortezomib in inducing p27Kip1 expression in CRC.^[38]^ Additionally, they have been investigated FOXM1 as a therapeutic target in CRC,^[39]^ and cyclooxygenase-2 inhibition in ovarian cancer.^[40]^ Their research also included findings on the leptin receptor and its role in the PI3K/AKT pathway in thyroid carcinoma^[41]^ and its association with poor outcomes in ovarian cancer.^[42]^ Finally, they contributed to the understanding of the phosphatidylinositol 3′-kinase/AKT pathway in diffuse large B-cell lymphoma survival.^[43]^ These studies exemplify their pivotal role in advancing cancer research in Saudi Arabia.

Among the most prolific authors in Saudi Arabia’s cancer research with global collaboration, Siraj and Abdul Khalid have emerged as leaders with 49 publications from 2020 to 2024. Affiliated with the King Faisal Specialist Hospital and Research Centre, he has made significant contributions to the field, often collaborating with prominent researchers such as Khawla S Al-Kuraya and Fouad Al-Dayel. His research spans a broad range of cancer types and focuses on the genetic and molecular mechanisms, prognostic biomarkers, and therapeutic targets. Notable studies include the identification of somatic MAP2K1 mutations in thyroid and CRCs, providing insights into their roles in tumorigenesis.^[44]^ He also contributed to research on the genetic heterogeneity of ovarian carcinoma, its metastatic evolution,^[45]^ and the differential expression of PD-L1 in primary and metastatic ovarian cancer, highlighting its clinical implications.^[46]^ His work on TERT promoter mutations established their role as predictors of metastasis in thyroid microcarcinomas.^[47]^ Additionally, he explored the impact of APC mutations in CRC, demonstrating the potential of tankyrase inhibitors to enhance chemotherapy efficacy.^[48]^ Other significant contributions include investigating the role of BRAFV600E mutations in upregulating PD-L1 expression in thyroid cancer and their prognostic relevance.^[49]^ Long-term follow-up studies on recurrence patterns in papillary thyroid cancer have provided valuable insights into disease progression.^[50]^ Collectively, Siraj and Abdul Khalid’s extensive research highlights their pivotal role in advancing cancer genetics, biomarker discovery, and targeted therapy development within Saudi Arabia’s global collaborative research framework.

A thematic map analysis of cancer research in Saudi Arabia highlights several research gaps across the full spectrum of cancer studies. Although foundational topics, such as breast cancer, gene expression, and chemotherapy, are well represented, there is limited emphasis on translational and clinical research to bridge laboratory findings and clinical applications. The declining focus on oxidative stress, inflammation, and chemotherapy-induced toxicities reflects a gap in addressing critical cancer pathophysiology and treatment challenges. Emerging technologies, such as AI and machine learning, show promise, but lack integration into real-world clinical applications for diagnosis and treatment planning. Additionally, there is insufficient focus on rare cancers, region-specific cancers, and advanced therapies, such as immunotherapy and precision oncology tailored to the genetic and environmental characteristics of Saudi Arabia. Longitudinal studies examining lifestyle, environmental, and genetic risk factors are also limited, which hinders the development of effective cancer prevention strategies. Addressing these gaps will help enhance the Kingdom’s cancer research landscape, foster innovation and targeted therapies, and improve healthcare outcomes.

Thematic analyses of cancer research in Saudi Arabia revealed notable differences when comparing data with international collaboration and independent Saudi contributions. In the first analysis, which included international collaborations, foundational topics such as breast cancer, gene expression, and screening were categorized as basic themes (Foundational Cancer Research) reflecting a global alignment on widely studied and fundamental cancer topics. In contrast, the second analysis, focused solely on Saudi-independent research, identified these as part of a Motor Theme (Comprehensive Cancer Research) highlighting their advanced development and central relevance within local priorities. In the first analysis, the Motor Theme emphasized molecular mechanisms and drug discovery, with keywords such as molecular docking and drug delivery indicative of the global influence in driving cutting-edge research. However, in the independent Saudi analysis, this theme broadens to include clinical and societal aspects such as COVID-19, awareness, and surgery, reflecting a practical orientation toward addressing domestic healthcare challenges. The Declining Theme, common to both analyses, highlights a reduced interest in oxidative stress and inflammation, but international collaboration includes broader related terms, such as diabetes and cardiotoxicity. In the first analysis, the Niche Theme integrates AI and imaging techniques, whereas independent analysis narrows its focus solely on AI applications, such as deep learning and machine learning. *Insights and recommendations*: The differences between the themes highlight the influence of international collaboration in driving high-tech molecular and experimental research, whereas Saudi-independent research focuses on practical, region-specific challenges, integrating cutting-edge technologies, such as AI, into clinical cancer management. To bridge these gaps and enhance the impact of SCR, fostering balanced collaboration that combines global expertise with local health priorities is crucial. Investments in advanced technologies, such as AI and molecular profiling, while maintaining a strong focus on clinical applications, can position Saudi Arabia as a leader in addressing regional cancer burden. Additionally, revitalizing declining themes through strategic funding and research initiatives can enhance our understanding of cancer etiology and potential treatments. These approaches will enable Saudi Arabia to achieve a comprehensive and globally competitive cancer-research ecosystem.

Trigram analysis revealed that squamous cell carcinoma (SCC) is a trending topic in SCR. SSC is a malignant tumor originating from squamous epithelial cells that commonly affect the oral cavity, skin, and cervix. Oral SCC is linked to risk factors such as tobacco use, alcohol consumption, and human papillomavirus (HPV) infection and requires early detection due to its aggressive nature. Skin SCC, primarily caused by chronic ultraviolet exposure, is the second most common skin cancer, and is highly treatable if identified early. Cervical SCC, which is largely associated with persistent HPV infection, highlights the importance of HPV vaccination and regular Papanicolaou smear screening. Although SCC share histological features across these sites, differences in etiology and progression necessitate site-specific prevention and management strategies. Emerging research on SCC in Saudi Arabia has focused on its epidemiology and incidence, oral squamous cell carcinoma, and cutaneous squamous cell carcinoma. Studies on SCC epidemiology highlight the importance of understanding the regional environmental and genetic factors that influence its incidence, with public health initiatives targeting early detection and prevention proposed as potential solutions. Research on oral squamous cell carcinoma emphasizes its clinical presentation, recurrence, and pathology, with hypotheses suggesting a significant role of HPV infection and the potential for advanced histopathological techniques to improve prognosis and management. Ongoing studies on cutaneous squamous cell carcinoma underscore the need to advance diagnostic and therapeutic approaches to enhance patient outcomes. These themes reflect the need for targeted prevention strategies and improved diagnostic and treatment methodologies tailored for the Saudi population.^[51–56]^

As shown in Figure 7, AML is a trending topic in SCR research. AML is a significant health concern in Saudi Arabia, ranking as one of the most common forms of leukemia and fifth most prevalent cancer in the country. It is more common in males, and the median age of the patients is lower than the global average. In recent years, the incidence of AML has increased, largely owing to improvements in diagnostic capabilities and reporting systems. Research has highlighted a variety of cytogenetic abnormalities including t(15;17), t(8;21), and complex cytogenetics. Molecular markers such as PML-RARA and NPM1 are associated with better overall survival, whereas mutations such as FLT3-ITD are associated with poorer outcomes. Treatment protocols in Saudi Arabia follow international guidelines, integrating advanced diagnostic tools, molecular risk stratification, and targeted therapies, with survival rates for certain subtypes comparable to those in developed countries. Emerging research themes focus on genetic and molecular profiling to refine risk stratification, prognostic models incorporating epigenetic markers, and the development of innovative therapies such as CAR-T cells, small molecule inhibitors, and precision medicine. Special attention is also given to non-intensive therapies and real-world treatment outcomes in elderly patients to address the unique challenges faced by this demographic. Despite this progress, several challenges remain, including the need for comprehensive epidemiological studies, unified national treatment protocols, and robust cancer registries to improve resource allocation and patient care.^[29,57–63]^ Expanding genetic and epigenetic research, integrating multi-omics data, and leveraging real-world evidence are crucial steps toward advancing personalized medicine and improving AML outcomes in Saudi Arabia.

## 5. Limitations

This study used the Scopus database to map SCR, which, while comprehensive, excludes publications indexed elsewhere. The search was limited to English-language articles and specific terms in titles, abstracts, and keywords, potentially omitting relevant studies with different phrases or in other languages. Standard bibliometric methods have been applied; however, as databases are continually updated, the findings may evolve. Future research could integrate Scopus with other databases, apply multimethod search strategies, and combine bibliometric analyses with systematic reviews for deeper insights. Author nationality and collaborator demographics were not assessed because of data limitations.

## 6. Conclusions

This study provides the first comprehensive bibliometric mapping of cancer research in Saudi Arabia, offering a multifaceted evaluation of trends, productivity, thematic focus, and collaboration patterns across decades. The analysis revealed a marked growth in research output, particularly between 2020 and 2024, highlighting the Kingdom’s intensified focus on oncology as a national health priority. These findings collectively indicate a shift from predominantly clinical to molecular and therapeutic cancer research, with increasing attention to precision medicine, AI, and diagnostics. Notably, prominent local researchers and institutions have contributed significantly to the shaping of independent research landscapes. However, Saudi Arabia’s overall research output remains modest compared with global standards, emphasizing the need for sustained international collaboration and capacity building. These insights are vital for guiding future investments in cancer R&D, aligning national health strategies with global research trends, and informing policymakers, funding agencies, and academic stakeholders. Although this study highlights an upward trajectory in cancer research productivity, it does not determine whether this growth has improved clinical outcomes such as patient survival. This limitation underscores the need to integrate bibliometric analyses with clinical and epidemiological data to assess real-world impacts. Future research should focus on mapping the translational impact of scholarly output, assessing the alignment of national research with the disease burden, and exploring interdisciplinary and cross-institutional collaboration models. In summary, this study establishes a strategic foundation for steering cancer research in Saudi Arabia and serves as a valuable resource for fostering innovation, closing knowledge gaps, and enhancing global scientific engagement in oncology.

## Author contributions

**Conceptualization:** Jobran M. Moshi, Siddig Ibrahim Abdelwahab, Manal Mohamed Elhassan Taha, Osama Albasheer, Saleh M. Abdullah, Abdullah Farasani, Mohammed A. Jeraiby, Humaid Obaid Al-Shamsi, Nizar A. Khamjan, Ahmad Assiri, Hussam M. Shubaily, Khaled A. Sahli.

**Data curation:** Siddig Ibrahim Abdelwahab, Manal Mohamed Elhassan Taha, Abdullah Farasani, Humaid Obaid Al-Shamsi, Ahlam Mohammed S. Hakami, Nizar A. Khamjan, Saeed Alshahrani, Hussam M. Shubaily, Khaled A. Sahli.

**Formal analysis:** Siddig Ibrahim Abdelwahab, Manal Mohamed Elhassan Taha, Humaid Obaid Al-Shamsi, Ahlam Mohammed S. Hakami, Nizar A. Khamjan.

**Funding acquisition:** Jobran M. Moshi, Siddig Ibrahim Abdelwahab, Manal Mohamed Elhassan Taha, Mohammed A. Jeraiby, Ahlam Mohammed S. Hakami, Nizar A. Khamjan.

**Investigation:** Siddig Ibrahim Abdelwahab, Manal Mohamed Elhassan Taha, Saleh M. Abdullah, Mohammed A. Jeraiby, Ahlam Mohammed S. Hakami, Nizar A. Khamjan.

**Methodology:** Siddig Ibrahim Abdelwahab, Manal Mohamed Elhassan Taha, Saleh M. Abdullah, Mohammed A. Jeraiby, Ahlam Mohammed S. Hakami, Nizar A. Khamjan, Saeed Alshahrani, Khaled A. Sahli.

**Project administration:** Siddig Ibrahim Abdelwahab, Manal Mohamed Elhassan Taha, Nizar A. Khamjan, Saeed Alshahrani.

**Resources:** Siddig Ibrahim Abdelwahab, Manal Mohamed Elhassan Taha, Nizar A. Khamjan, Ahmad Assiri.

**Software:** Siddig Ibrahim Abdelwahab, Manal Mohamed Elhassan Taha, Nizar A. Khamjan, Ahmad Assiri.

**Supervision:** Jobran M. Moshi, Siddig Ibrahim Abdelwahab, Manal Mohamed Elhassan Taha, Ahmad Assiri.

**Validation:** Jobran M. Moshi, Siddig Ibrahim Abdelwahab, Manal Mohamed Elhassan Taha, Abdullah Farasani, Khaled A. Sahli.

**Visualization:** Jobran M. Moshi, Siddig Ibrahim Abdelwahab, Manal Mohamed Elhassan Taha, Osama Albasheer, Saleh M. Abdullah, Abdullah Farasani, Mohammed A. Jeraiby, Humaid Obaid Al-Shamsi, Saeed Alshahrani, Khaled A. Sahli.

**Writing – original draft:** Jobran M. Moshi, Siddig Ibrahim Abdelwahab, Manal Mohamed Elhassan Taha, Osama Albasheer, Saleh M. Abdullah, Abdullah Farasani, Mohammed A. Jeraiby, Humaid Obaid Al-Shamsi, Ahlam Mohammed S. Hakami, Saeed Alshahrani, Ahmad Assiri, Hussam M. Shubaily, Khaled A. Sahli.

**Writing – review & editing:** Jobran M. Moshi, Siddig Ibrahim Abdelwahab, Manal Mohamed Elhassan Taha, Osama Albasheer, Saleh M. Abdullah, Abdullah Farasani, Mohammed A. Jeraiby, Humaid Obaid Al-Shamsi, Hussam M. Shubaily, Khaled A. Sahli.

## Supplementary Material
