# Supplementary material for: Cancer research in Saudi Arabia: A cross-sectional mapping study of historical growth, thematic analysis, collaboration patterns, and future directions
Source: Medicine (Baltimore). 2025 Sep 26;104(39):e44757. doi: 10.1097/MD.0000000000044757 (PMC13593311; doi:10.1097/MD.0000000000044757)
Supplement: Supplementary file 1 [file medi-104-e44757-s001.docx]

"cancer" OR "neoplasm" OR leukemia OR "tumour" OR "tumor" OR "carcinoma" OR "adenocarcinoma" OR "leukemia" OR "leukaemia" OR "sarcoma" OR "lymphoma" OR "malignant" OR carcinogenesis OR "oncology" OR "metastasis" OR "oncogene" OR "chemotherapy" OR "Lung cancer" OR "Colorectal cancer" OR "Liver cancer" OR "Prostate cancer" OR "Leukemia" OR "Hematological malignancies" OR "Skin cancer" OR "Gastrointestinal cancer" OR "Thyroid cancer" OR "Pediatric cancer"

7,162,715 documents found

AND AFFILCOUNTRY ( "Saudi Arabia" ) )

40,180 document results

( LIMIT-TO ( DOCTYPE , "ar" ) ) AND ( LIMIT-TO ( LANGUAGE , "English" ) )

32,193 documents found

AND ( LIMIT-TO ( PUBYEAR , 2020 ) OR LIMIT-TO ( PUBYEAR , 2021 ) OR LIMIT-TO ( PUBYEAR , 2022 ) OR LIMIT-TO ( PUBYEAR , 2023 ) OR LIMIT-TO ( PUBYEAR , 2024 ) AND ( LIMIT-TO ( DOCTYPE , "ar" ) ) AND ( LIMIT-TO ( LANGUAGE , "English" ) )

18,415 document results
